# Implicit and explicit changes in body satisfaction evoked by body size illusions: Implications for eating disorder vulnerability in women

**DOI:** 10.1371/journal.pone.0199426

**Published:** 2018-06-21

**Authors:** Catherine Preston, H. Henrik Ehrsson

**Affiliations:** 1 Psychology Department, University of York, York, United Kingdom; 2 Department of Neuroscience, Karolinska Institutet, Stockholm, Sweden; University G d'Annunzio, ITALY

## Abstract

Dissatisfaction with one’s body is a widespread issue in modern society and has been linked to vulnerability for developing eating disorders. Recent studies have demonstrated a direct relationship between body perception and body satisfaction by manipulating perceived body size using multisensory body illusions. However, how these body size illusions influence implicit affective experience has not previously been examined. The current experiment used an established full-body ownership illusion paradigm to induce feelings of illusory obesity in male and female participants. The effects of illusory obesity on explicit and implicit body satisfaction were measured in naïve participants across two separate experiments. In terms of explicit measures, owning an obese body decreased body satisfaction, and owning a slimmer body increased body satisfaction in females but not in males. However, implicit feelings regarding the body were only influenced by the synchrony of the touch and not the size of the body in the illusion. These results suggest that implicit and explicit affective experiences of the body may be mediated by different factors. In addition, these findings may have clinical implications because both implicit and explicit changes in affective experience of the body were related to behaviours and thoughts associated with disordered eating in a non-clinical sample.

## Introduction

In today’s modern society, concerns regarding the body are widespread. Negative feelings regarding the body are thought to be related to the development of eating disorders [1], which may be associated with long-term physical [2] and psychological problems [3], as well as high relapse [4] and mortality [5] rates compared with other psychiatric disorders. It has long been thought that females have higher levels of dissatisfaction regarding their bodies than males [6–8]; however, recent research provides evidence that body concerns in men are also increasing [9,10].

A key factor suggested to contribute to body (dis)satisfaction and ultimately the development of eating disorders and eating disorder symptoms is the internalisation of social ideals regarding the body [11–13]. In modern western society, there is a strong emphasis on thinness, particularly for women, with the social definition of an ideal body being unattainable for most individuals. Deviation from this social ideal may thus elicit negative feelings regarding the body, as well as dieting, disordered eating and other unhealthy weight control behaviour [11]. According to this theory, the larger the actual body size is (the further away from the ideal body shape), the worse one feels about oneself [11,14,15]. Many studies have shown a relationship between body size and body dissatisfaction, particularly for women who seem to have stronger negative responses to equivalent increases in body size (correlational study) [6,16]. This association between body size and body satisfaction was present not only in terms of actual body size but also for perceived body size when individuals were asked how big their body feels [17]. This relationship may be particularly important for clinical eating disorder patients who are thought to have an inaccurate experience of their own body [18–24], and although body perception is only one aspect of these complex disorders, it is linked to poor prognosis [21,24]. However, until recently, empirical evidence for a direct link between how we perceive (body size) and how we feel about our body (body satisfaction) has remained elusive.

Multisensory body ownership illusions enable transient illusory modulations of the perceptual experience of the body [25–27]. These illusions work on principles of multisensory integration to elicit feelings of ownership over fake or stranger’s bodies/ body parts. The most well studied multisensory body ownership illusion, the rubber hand illusion, induces feelings of ownership over a fake hand by touching the seen fake hand at the same time and in the same place as touches delivered to the real hand, which is hidden from view [28]. This visual-tactile synchronicity (matching of vision and touch) leads to multisensory integration, such that the brain interprets the vision and touch as a single event. Therefore, the fake hand feels like it is the participant’s real hand provided the embodied hand meets constraints related to appearance [29,30], orientation [31–34] and spatial location [35,36]. However, touching the real and fake hands asynchronously means that vision and touch are not integrated and illusory ownership does not occur.

Adapting this illusion to the entire body using a virtual reality headset enables the induction of a full body ownership illusion in which participants feel as though a different (mannequin’s or stranger’s) body is their own body [37]. Modulating the appearance of the body, or part of the body, may subsequently induce illusory modulation to the perceived size and shape of the actual body (part) [38–43], thus enabling the experimental modulation of body size/shape. In our previous studies, we determined that illusory ownership over a mannequin body that was 15% slimmer than the participant increased their self-reported body satisfaction [44], whereas ownership over an obese stranger’s body reduced body satisfaction [45]. Interestingly, decreases in body satisfaction from illusory obesity were greater in females than males, whereas increases in body satisfaction as a result of feeling slimmer did not differ between the sexes [44]. Notably, ownership over a slim stranger’s body did not increase body satisfaction [45] in the same way as ownership over a slimmer mannequin [44]. This discrepancy between the effect on the body satisfaction of ownership over real and mannequin bodies is likely to be a result of mannequins being designed specifically to encapsulate unattainable social ideals. Average shop mannequins portray unhealthy body sizes and shapes, particularly for females [46,47]. Our previous study also systematically ensured that the mannequin body was 15% slimmer than the participant’s body [44], such that ownership over a slim mannequin is likely to reflect these unattainable social ideals more so than a slim real model.

Importantly, changes in body satisfaction in response to these body size illusions have also been linked to non-clinical eating disorder thoughts and behaviours in healthy individuals [44]. In this study, these cognitive-behaviour characteristics correspond to scores on the eating disorder examination questionnaire, a clinical measure of eating disorder symptoms, which fall below established clinical cut-offs [44,45]. Interestingly, we have previously shown that greater changes in body satisfaction induced by a full-body ownership illusion in healthy individuals were associated with higher cognitive-behavioural characteristics associated with eating disorders [44]. However, our earlier findings measured body satisfaction changes only through explicit reports of current body satisfaction, which are vulnerable to demand characteristics, and we only investigated the effects of illusory obesity during functional magnetic resonance imaging (fMRI) using a single questionnaire item response [45]. Moreover, implicit emotional mechanisms are thought to play an important role in disordered eating pathology [48] and have been argued to be a closer link to true emotional states, with implicit emotions and attitudes being largely overlooked as a target for most present-day therapies, which primarily focus on explicit responses [49]. Therefore, the current experiment aims to investigate whether illusory ownership of an obese body influences implicit attitudes towards the real body in males and females. It is predicted that implicit body satisfaction will demonstrate the same pattern as for explicit measures; specifically, ownership of an obese body will decrease body satisfaction in females but not in males. Illusory ownership of a slim stranger’s body was included as a control condition in the current study, with the prediction that in the slim conditions, satisfaction towards the body would not be influenced on an implicit or explicit level for either sex.

In the present experiments, participants were subjected to an illusory experience of ownership over sex-matched bodies of slim and obese strangers. Explicit (experiment one) and implicit (experiment two) measures of body satisfaction were obtained at baseline and after each (illusion and control) condition. Explicit measures were obtained with the Body Image States Scale [50] designed to capture body satisfaction at a particular moment in time, and implicit measures were obtained using an adaptation of the implicit association task (IAT) [51]. The IAT was originally designed to capture implicit social attitudes, which may be subject to social desirability bias with explicit responses or of which we are not explicitly conscious [45]. The IAT has subsequently been adapted to capture implicit attitudes regarding the self, such as self-esteem [52,53] and attitudes towards the body [52].

The current study used a body satisfaction IAT paradigm (adapted from [52]) to measure implicit associations between the self and attractiveness and whether these associations may be modulated as a result of the transient illusory experience of being obese. A previous study by Gumble and Carels using an IAT to measure body attitudes [52] indicated that body size, as well as eating disorder symptoms were related to associations between the self and attractiveness, with larger individuals and individuals with more eating disorder symptoms demonstrating reduced associations between self and attractiveness. Gumble and Carles also identified a significant correlation between IAT scores and explicit feelings regarding the body, which supports the assumption that implicit and explicit body attitudes are related. However, differences between the sexes for the IAT score in relation to body size were not identified in the previous study. Moreover, they did not identify sex differences with their explicit measures, in contrast to other studies [6,8]. Nevertheless, to complement explicit measures of body satisfaction, we used the IAT to provide implicit behavioural evidence of illusion-induced changes in attitudes towards one’s body.

In the current study, explicit body satisfaction is measured using the Body Image States Scale (BISS). The BISS is a six-item scale that assesses how participants explicitly feel about their body *right now*, thus representing an appropriate measure to capture transient changes in body satisfaction [50]. The BISS has been shown to be sensitive to both positive (e.g., party compliments) and negative (e.g., exposure to magazine models) contexts [50]. Furthermore, the BISS was also used in our previous study to capture positive increases in body satisfaction as a result of illusory ownership over a slimmer mannequin body [44]. BISS scores relate to other validated trait measures of body satisfaction in both males and females and have been shown to demonstrate sex differences (females exhibit significantly lower body satisfaction than males) [50] as shown by the majority of previous studies using other measures [e.g., 6,8].

In summary, our study will employ a full-body ownership illusion with obese and slim bodies to re-examine the link between body (size) perception and body satisfaction. More specifically, we will examine whether implicit responses on the IAT may be modulated purely by the illusion of ownership over an obese body in both males and females and whether implicit body satisfaction demonstrates the same sex differences as reported using explicit measures of body satisfaction (BISS). As previously discussed, it is predicted that illusory ownership over an obese body will reduce both explicit and implicit measures of body satisfaction in female but not male healthy participants. In line with our previous findings [45], it is also predicted that this reduction in body satisfaction will be related to non-clinical levels of cognitive-behavioural eating disorder characteristics.

## Materials and methods

### Experiment one

#### Participants

Forty participants (20 males and 20 females) with a mean age of 27 years (range 19–41) participated in the study. All participants provided written informed consent; the experiment was conducted in accordance with the Declaration of Helsinki and was approved by The Regional Ethical Review Board of Stockholm (Regionala etikprövningsnämnden i Stockholm; www.epn.se). All participants were pre-screened prior to inclusion in the experiment using a similar protocol to previous experiments [44,45], such that only individuals who did not meet our exclusion criteria were recruited.

The exclusion criteria consisted of the following:

- Current psychiatric conditions as assessed using the Mini International Neuropsychiatric Interview screen [54].
- A global score greater than 2.8 (or +4 on any of the subscales) on the Eating Disorder Examination Questionnaire (EDE-Q) (refer to subsequent description) indicating the possibility of a clinical eating disorder [55].
- Previous history of psychiatric or neurological disorders assessed by self-report.
- Living in Sweden for less than 12 months [44] assessed by self-report.
- BMI greater than our obese sex matched models (refer to subsequent description) assessed by weight and height information collected during the screening process, which was then verified when participants arrived for the experiment.

Although some individuals had BMIs that suggested the participants were overweight, all participants’ BMIs were lower than the BMI of our obese model. Participants also completed the Rosenberg Self-Esteem Scale [56] as a general control measure of self-attitudes (as opposed to attitudes specifically directed towards the body).

#### Materials

During the experiment, the participants lay on a single bed wearing a set of head-mounted displays (HMDs) (Cybermind Visette45, Cybermind Interactive, Maastricht, the Netherlands) with a field of view of 45° and a display resolution of 1280×1024. Through the HMDs, video images were presented of sex-matched slim and obese bodies, and these videos were the same as the videos used for a previous neuroimaging experiment [45] adapted for the specific resolution of these HMDs. A pillow was used to prop up the participant’s head to enable them to look down as if viewing their own body without significant discomfort.

**Fig 1:**
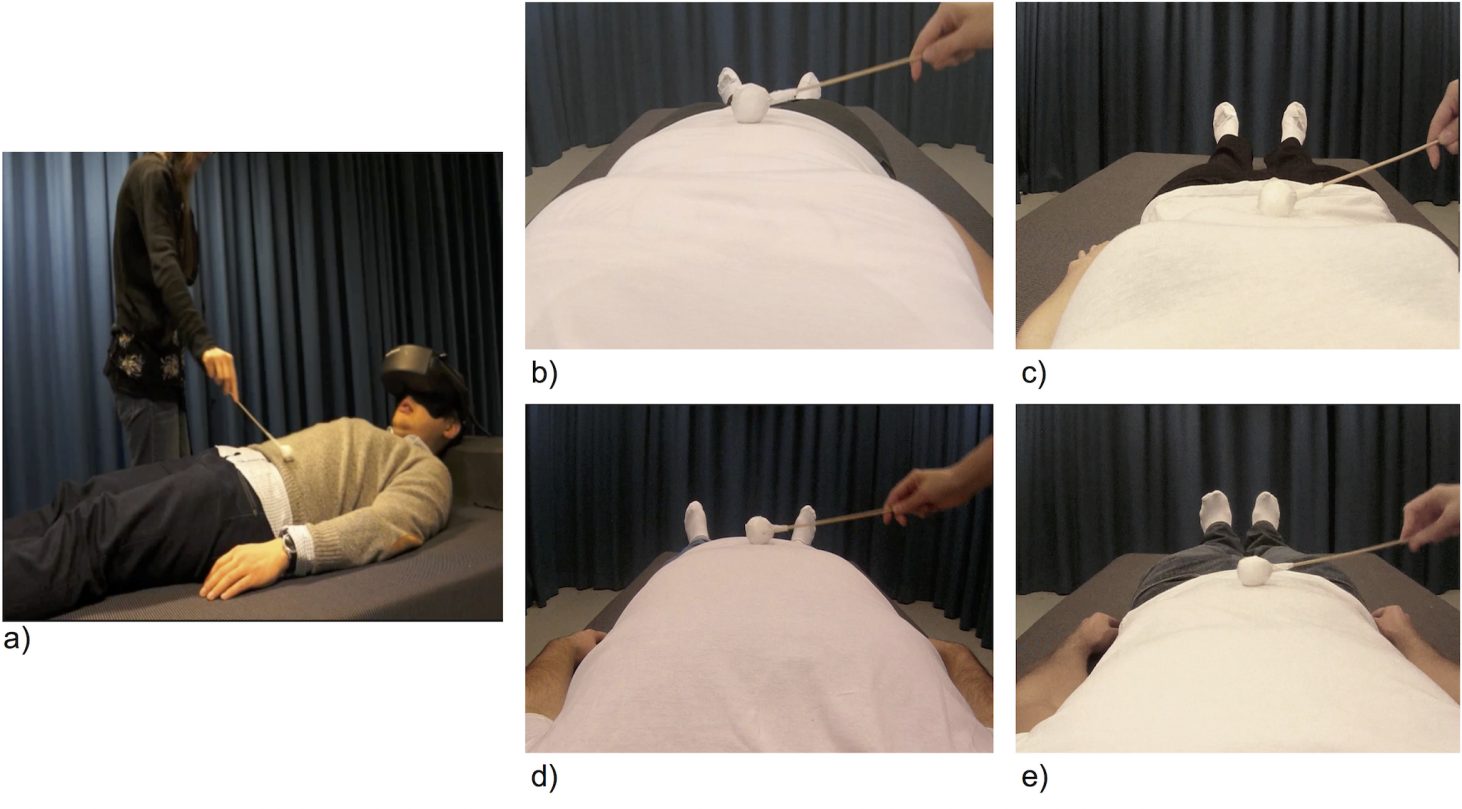
Experimental set-up. Participants lay on the bed and tilted their head forward wearing the head mounted displays (HMDs) while the experimenter touched their torso with a small white ball attached to a stick (a). The touches were synchronous or asynchronous (approximately 1000 ms delay) with touches delivered to the model in the video (viewed through the head mounted display). Still images from the videos of the different body types being touched with the ball on the stick: female obese (b), female slim (c), male obese (d), male slim (e).

To present 3D images of realistic slim and obese bodies, pre-recorded videos were produced by filming the bodies of real (slim and obese) models from a first-person perspective (created for [39]). In accordance with traditional views of the ideal body type for males [9,13,57], the slim male model had a muscular physique and a BMI of 20.4. The obese male model had a BMI of 36. In line with traditional theories of ideal body type in females [11,13], the slim female model was selected based on body size and had a BMI of 18.4, whereas the obese female model had a BMI of 32.3 (Fig 1). During filming, the models were instructed to lie still on a bed with their arms at their sides. Two identical cameras (CamOne Infinity HD, resolution 1920 × 1080, Touratech AG, Germany) were placed on a stand immediately above their eyes, capturing images of their body, while the experimenter delivered tactile stimuli to their torso using a stick (300 mm in length) attached to a white polystyrene sphere (30 mm diameter). The two videos were subsequently synchronised using Final Cut Pro 7 (Apple) with the recordings from the left and right cameras (corresponding to the viewpoints of the left and right eye, respectively) placed side by side in a single frame corresponding to the display resolution of the HMDs (1280 × 1024 pixels).

#### Measures

Body satisfaction was measured using the Body Image States Scale (BISS). The BISS is a six-item scale designed to measure body satisfaction at a particular instance in time. Responses are made on a nine-point Likert-type scale with three questions anchored negative to positive and three questions anchored positive to negative (reverse scoring). The BISS correlates with other body satisfaction measures and demonstrates internal consistency and construct validity [50]. Higher BISS scores represent higher body satisfaction.

Cognitive-behavioural eating disorder characteristics were measured using the Eating Disorder Examination Questionnaire (EDE-Q) 6.0, which has good internal consistency and reliability for women [58] and men [59–61]. The questionnaire consists of 28 items rated on a seven-point Likert scale, with the exception of six items that assessed the frequency of behaviour. The questionnaire may be divided into four subscales (dietary restraint, eating concern, weight concern, and shape concern) or comprise a single global measure. Because the global score was used to define the clinical cut-off [55], it was this score that was used for all subsequent analyses (for a breakdown of the different subscales for both experiments one and two, refer to S1 Table).

The subjective experience of the illusion was measured using an illusion questionnaire (all questions were adopted from our previous study [44] and were comparable to previous similar research [42,62]). This questionnaire consisted of seven statements, which the participants had to rate using a seven-point Likert scale, +3 (strongly agree) to −3 (strongly disagree) (S2 Table). Two statements were designed to capture the strength of the basic ownership illusion and two control questions were included to control for task compliance and suggestibility effects; two additional statements directly assessed “feeling fatter” or “feeling slimmer”, and one statement assessed perceived attractiveness.

#### Procedure

During the experiment, the participants lay with their heads tilted (approximately 25°) on a bed while wearing the HMDs. The pre-recorded videos were presented to the participants through the HMDs such that the body of the model appeared in the same position and from the same perspective as if directly viewing their actual body. The experiment was conducted as two separate parts; the first part measured body satisfaction, and the second part measured the body ownership (illusion). For the first part of the experiment, each participant participated in one trial of all four conditions: viewing a slim body that was touched by the experimenter in synchrony with touches delivered to the participant’s own body, viewing an obese body touched in synchrony with touches delivered to the participant’s body, viewing a slim body that was touched asynchronously with touches to the participant’s own body, and viewing an obese body that was touched asynchronously with touches to the participant’s own body. Each trial comprised 30 seconds of playing 3D video images of obese or slim bodies being touched 18 times equally divided between the left, right, and centre of the torso (see above), while the same touches where delivered to the participant. Thirty-second trials were used to make it equivalent to a previous fMRI study, which demonstrated that 30 seconds of repeated visuo-tactile stimulation was sufficient to experience the illusion and record changes in body satisfaction [45]. The touches were delivered by the experimenter using an identical stick to that used to create the videos (300 mm in length with a white 30 mm diameter polystyrene sphere). The timing of the touches in the videos was controlled by audio cues that indicated when and where to touch, which were played to the experimenter during filming. The same audio track also cued the experimenter to deliver the touches during the experiment and was synchronised with the video images (synchronous trials) or delayed by 1000 ms (asynchronous trials). The tones were played to the experimenter via headphones such that they were clear to the experimenter yet inaudible to the participants during the experiment. Each touch covered approximately 5 cm of the body, and the touches were delivered at approximately 10 cm/sec (500 ms per touch). At the beginning of the experiment and after each trial, the participants completed the Body Image State Scale (BISS). The questions for the BISS were presented in a random order through the HMDs, and responses were provided verbally and were entered into the computer by the experimenter. To protect the confidentiality of participant responses, the experimenter could not see the questions, and reverse scoring was implemented (refer to scale information above) such that the experimenter could not tell whether the responses were positive or negative. The participants were informed of these measures so that they were aware of the confidentiality of their responses.

**Fig 2:**
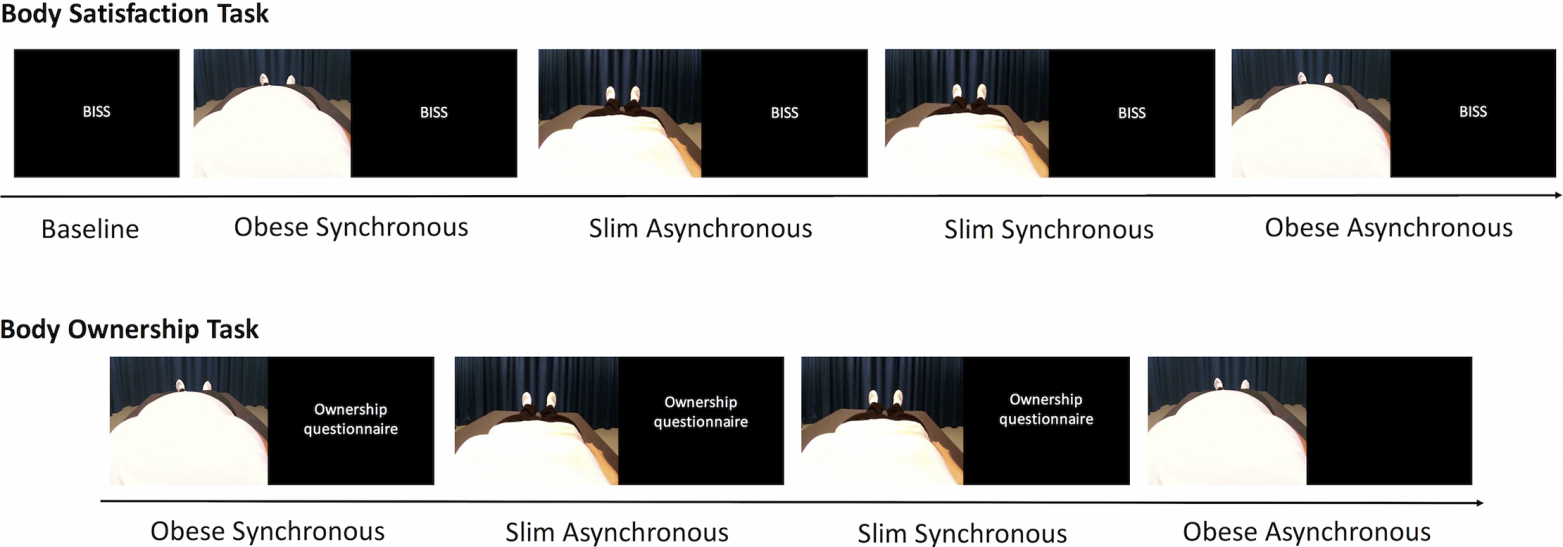
Schematic representation of experiment. The participants always completed the trials that measured body satisfaction first (top image) followed by body ownership (bottom image). Participants completed the Body Image States Scale (BISS) at baseline and after each experimental trial for the body satisfaction task. The illusion questionnaire (which measured body ownership) was completed after each experimental trial of the body ownership task. The order of the trials alternated between synchronous and asynchronous and was identical for both tasks for a single participant; however, it was counterbalanced across participants. This schematic shows an example trial order.

For the second part of the experiment, the participants again participated in one trial each of all four conditions. After each trial, the participants rated their subjective experience of the illusion using the illusion questionnaire (refer to previous description and S2 Table). All items were presented to the participant in a random order via the HMDs with (numeric) responses verbally provided to the experimenter. The order of the trials was identical for the first and second parts of the experiment; however, it was counterbalanced across participants with the prerequisite that trials alternated between synchronous and asynchronous to make it directly comparable to our previous fMRI experiment [44]. For a schematic representation of the experiment, refer to Fig 2.

### Experiment two

#### Participants

Sixty-four naïve participants (32 males and 32 females) with a mean age of 26 years (range 18–39) participated in the study. We recruited naïve participants for this study to reduce the effects of expectations and demand characteristics. All participants provided written informed consent; the experiment was conducted in accordance with the Declaration of Helsinki and was approved by The Regional Ethical Review Board of Stockholm (Regionala etikprövningsnämnden i Stockholm; www.epn.se). Identical screening procedures were implemented as for experiment one (refer to previous description). A greater number of participants was used for experiment two because of the IAT measure, which generally requires larger samples [53]. A previous rubber hand illusion study that employed the IAT to measure racial bias used a sample size of 27. Based on this study, we recruited 64 participants (32 females and 32 males) because one of our main aims was to assess potential differences in male and female responses and this specific number fit counterbalancing of both body size conditions and IAT presentation (compatible or incompatible presented first on the left or right of the screen).

#### Materials

The same materials were used as for experiment one with the exception of the addition of two button boxes (one for each hand) to record the IAT responses. These button boxes were constructed in house and corresponded to keyboard presses of ‘U’ and ‘I’ keys for left and right responses, respectively.

#### Procedure

Experiment two involved an identical procedure as that for experiment one with the exception that instead of participants completing the BISS, the IAT was completed at the beginning of the experiment and after each trial for the body satisfaction task (Fig 2).

#### Implicit association task

**Fig 3:**
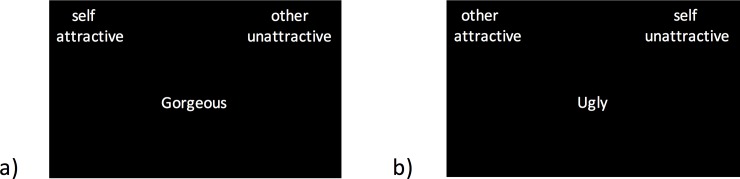
Schematic of body satisfaction implicit association task (IAT). A) Example compatible trial, in which the categories of self and attractive are paired (other and unattractive are also paired). B) Example incompatible trial, in which the categories of self and unattractive are paired (other and attractive are also paired). Words that belong to one of the four categories appeared in the centre of the screen, and participants had to respond as to whether the word in the centre belonged to a category on the left or right.

The body satisfaction IAT was based on a previous study [52], in which participants were instructed to categorise words that appeared in the centre of the screen as attractive or unattractive or self or other. Some words from the original study were replaced because they were not suitable for a multinational sample (e.g., homey). Prior to the experiment, the participants underwent a short practice session for the IAT paradigm on a laptop computer while seated at a table, and responses were selected with button boxes. This session familiarised the participants with the task and how to respond. For the first six practice trials, two different categories appeared on the screen, one category on the left and one category on the right (e.g., attractive and unattractive). Words subsequently appeared in the centre of the screen that belonged to one of the two categories, and the participants had to identify whether the central word belonged to the category on the left or right by pressing the appropriate button box. The participants were instructed to respond as quickly and accurately as possible. The participants subsequently completed the same task using the other two categories (e.g., self and other) for an additional six practice trials. In the next 12 trials, two categories appeared on each side of the screen (e.g., self/attractive and other/unattractive). The words that appeared in the centre of the screen belonged to one of the four categories, and the participants had to indicate whether each word belonged to one of the categories on the left or right by pressing the appropriate button box. For the final practice block, which also consisted of 12 trials, all four categories appeared again but in different pairs (e.g., self/unattractive and other/attractive). Following the practice session, the participants were asked if they understood the task and all words (all participants were fluent in English; however, they were not all native English speakers) prior to putting on the HMDs and laying on the bed. In this experimental set-up, the participants completed an additional 12 practice trials with different stimuli (good/bad, flower/insect) to verify that they could read the stimuli through the HMDs and were comfortable responding with the button boxes in a supine position. Refer to supplementary S3 Table for the list of words used in the IAT and Fig 3 for a schematic of typical trials.

Following the practice trials, the participants participated in two 60-trial blocks of the IAT. In one block, the self and attractive categories were paired on the same side of the screen (along with other and unattractive), and in the second block, the self and unattractive categories were paired on the same side of the screen (along with other and attractive). The same two blocks were subsequently repeated in the same order after each experimental condition. The order in which the blocks with the different pairs were presented, along with the side of the screen each pair was presented on, was counterbalanced across the participants. As for experiment one, the participants subsequently participated in one trial of each condition, following which they responded to the illusion questionnaire (refer to previous description and S2 Table).

#### Data analysis

Prior to analysis, all data were tested for the normality of the distribution using Shapiro-Wilk tests. When violations of normality were identified by significant Shapiro-Wilk statistics, non-parametric tests were used, specifically Wilcoxon signed rank tests for within tests and Mann-Whitney U tests for between tests. Non-parametric correlations were analysed using Spearman’s Rho. When there were no violations of normality, parametric statistics were used, specifically mixed ANCOVA, paired samples, independent samples, and one-sample t-tests. Parametric correlations were measured using the Pearson’s r product-moment correlation coefficient.

**Descriptives**

Medians and interquartile ranges are reported for non-parametric data, whereas means and standard deviations/standard errors are reported for parametric data.

**Body ownership illusion**

Illusion scores for both experiments were calculated by averaging across the two illusion questions for each condition (in line with previous studies, e.g., [38,44]). Similarly, control scores were calculated by averaging across the two control questions. We statistically assessed the difference for the illusion and control scores between the synchronous and asynchronous conditions for both body sizes independently and examined between body sizes with the synchronous conditions only. This analysis was also performed independently for males and females (reported in S1 Text), as well as directly testing for differences in the illusion score for the synchronous conditions between males and females (reported in the main text).

**Feelings of Fatness/Thinness**

To explore potential subjective experiences of “fatness” and “slimness” for both experiments one and two during each condition, we statistically assessed the difference between the synchronous and asynchronous responses to the questions “my body felt fatter than usual” and “my body felt thinner than usual” for obese and slim body sizes, respectively. This analysis was completed across the entire sample and for males and females independently (the latter reported in the S1 Text). Differences in the responses between the sexes were also examined by directly comparing the scores between males and females.

**Explicit body satisfaction**

Explicit body satisfaction was calculated by obtaining a mean of all six items of the BISS at baseline and after each condition after reverse scoring. The BISS scores following each condition were subsequently subtracted from the baseline scores and entered into a mixed ANCOVA, in which the between factor was the sex of the participant to assess differences in the responses between males and females and the covariate was EDE-Q score. The EDE-Q was used as a covariate because of the a prior hypothesis that it would be associated with body satisfaction responses based on previous findings [45]. BMI was not included as a covariate because of previous findings that body satisfaction change was not related to individual BMI (also supported in the current results).

To directly test our main hypothesis that illusory ownership over an obese body would result in a change in body satisfaction for females but not for males, the ANCOVA was followed up with planned comparisons between body satisfaction in the synchronous compared to asynchronous conditions for both obese and slim body sizes for females and males independently. Our hypothesised null results between the obese synchronous and obese asynchronous conditions in males was further examined by calculating a Bayes factor (B value). Bayes factors provide statistical support for the null hypothesis irrespective of the sample size [63]. A Bayes factor less than 1/3 indicates evidence for the null hypothesis, whereas a Bayes factor greater than 3 indicates evidence for the alternative hypothesis [63]. The estimated effect size used to calculate the Bayes factor was derived from our observations with females. We also examined changes from baseline for each condition using one sampled tests, and the relationship between body satisfaction change (BISS synchronous minus BISS asynchronous) and eating disorder characteristics (EDE-Q score) was examined using bivariate correlations.

**Correlating feelings of “Fatness/Thinness” and explicit body satisfaction**

To directly examine the relationship between feelings of being fatter or thinner and changes in body satisfaction, subjective increases in fatness ratings were calculated by subtracting the agreement scores with the question “my body felt fatter than usual” in the asynchronous conditions from the synchronous conditions for the obese body size conditions. Similarly, illusory decreases in subjective slimness experiences were calculated by subtracting the agreement scores with the question “my body felt thinner than usual” in the asynchronous conditions from the synchronous conditions for the and slim body size conditions. These scores were subsequently correlated with the change in the body satisfaction score for the same size body (synchronous minus asynchronous, refer to previous description) for the whole sample.

**Implicit body satisfaction**

Implicit body satisfaction: Trials with reaction times longer than 3000 ms or shorter than 300 ms were removed along with the first two trials from each block and all incorrect responses. The mean percentage of removed trials (including incorrect responses and trials that fell outside of the 300 ms to 3000 ms range) was 3.1% (range .34% to 10.34%), which falls within typical IAT error rates [64]. The modified d scores were subsequently calculated for each participant in each condition. Our d scores were calculated accordingly: the latencies from compatible trials (self-paired with attractive and other paired with unattractive) subtracted from the latencies from the incompatible trials (self-paired with unattractive and other paired with attractive) with the sum divided by the inclusive standard deviation. The d scores for each experimental condition were subsequently subtracted from baseline so that negative scores represented relative decreases from baseline and positive scores represented relative increases. Because the cognitive-behavioural eating disorder characteristics and sex of the participants were predicted to impact the results [44,45], sex was examined as a between subjects variable and the EDE-Q score was examined as a covariate (as for experiment one). Therefore, the data were entered into a 2x2 mixed ANCOVA.

To directly test our main hypothesis that illusory ownership over an obese body but not a slim body would result in a change in body satisfaction for females, comparisons were conducted that examined body satisfaction in the synchronous compared to asynchronous conditions for both body sizes for males and females independently. We also statistically examined the changes from baseline using one-sample tests. We examined the relationship between the eating disorder characteristics (EDE-Q) and implicit body satisfaction change (d-score synchronous minus d-score asynchronous) for both males and females independently using bivariate correlations. Other non-hypothesised significant interactions were followed up using post hoc paired sample tests and correlations.

**Additional correlations**

Correlations of other measures (including the EDE-Q subscales, BMI and self-esteem) with both implicit and explicit body satisfaction changes are reported in S4 Table and S5 Table. No correlations of interest were identified.

**Correcting for multiple comparisons**

Our main hypotheses were examined using a priori planned comparisons, and all other comparisons were corrected using false discovery rates. Effect sizes are reported as Cohen d for t-tests, partial eta squared for F tests, and r values [65] for non-parametric (Wilcoxon and Mann-Whitney U) tests for which small medium and large effect sizes are equivalent to Cohen d.

## Results

### Experiment one

#### Descriptives

There were no significant differences between the male and female participants for any of the demographic (age and BMI) or attitudinal (EDE-Q and SE) measures assessed (Table 1).

**Table 1:** Participant demographics experiment one.

Measure	Total	Male	Female	T statistic	P value
Age	27 years (5.5) [19–41]	27 years (4.2) [21–34]	28 years (6.7) [19–41]	.05	.59
BMI	22.7 (2.8) [18.5–29.7]	23.0 (2.4) [19.9–28.8]	22.3 (3.7) [18.5–29.7]	.72	.48
SE	22.5 (4.9) [8–30]	22.5 (6.0) [16–30]	22.6 (6.0) [8–30]	.095	.93
EDE-Q	.35 (.11 - .67) ^a^ [0–2.1]	.32 (.11 - .79) ^a^ [0–1.7]	.42 (.11 - .68) ^a^ [0–2.1]	.81^b^	.43

The table presents the means with standard deviations in parentheses and ranges in square brackets for participant age, body mass index (BMI) self-esteem measured by the Rosenberg Self-Esteem Scale (SE) and cognitive-behavioural eating disorder characteristics measured by the eating-disorder examination questionnaire (EDE-Q) score.

^a^Median and inter quartile range in parentheses

^b^Mann-Whitney U statistic

#### Body ownership illusion

**Fig 4:**
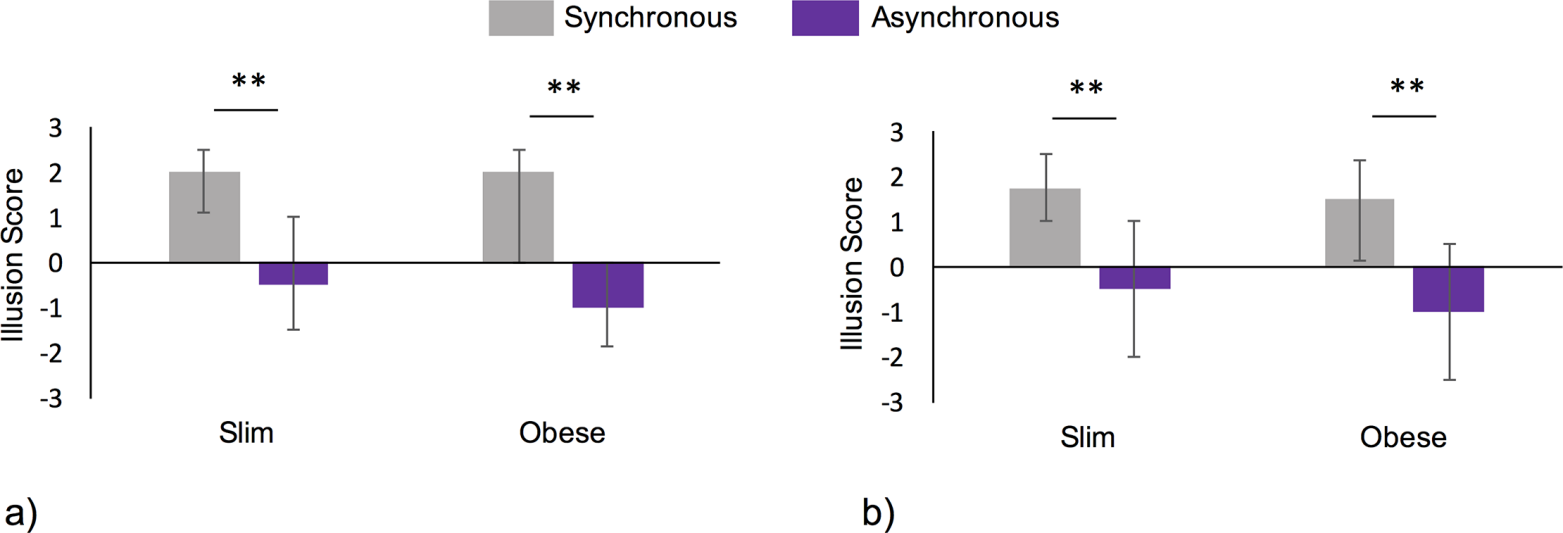
Illusion scores for experiments one and two. Agreement with illusion relevant questionnaire items was higher following synchronous touch (light grey bars) than following asynchronous touch (mauve bars) for both the slim and obese bodies in experiment one (a) and experiment two (b). No significant difference in illusion strength was identified between the two body sizes for either experiment. Bars represent median scores and error bars depict inter-quartile ranges. ** = p < .001.

The questionnaire data (illusion composite score) were ordinal, and both body size synchronous conditions were not normally distributed (max Shapiro-Wilk test statistic = .94, p = .03); thus, they were analysed using non-parametric tests. To determine the effect of the synchrony of touch for each body size condition, Wilcoxon signed rank tests were conducted between the synchronous and asynchronous conditions for both the obese and slim body types. A significant effect of synchrony was identified with the obese body (z  =   -4.34, p < .001, r = .50) having higher illusion ratings for the synchronous (median   =  2.0) than asynchronous (median   =  −1.0) touch. The same was true of the slim body (z  =   -4.7, p < .001, r = .53, synchronous median =  2.0, asynchronous median   =  −.5) (Fig 4A). However, equivalent tests on the control scores indicated there was no effect of synchrony with the obese (z   =  -.64, p  = .653, r = .07) or slim (z   =  -.28, p  = .78, r = .03) bodies. Thus, in terms of the basic body ownership illusion, our paradigm worked as expected [44,45], with well-matched levels of ownership illusion also identified across body types and sex of the participants (see S1 Text).

#### Feelings of Fatness/Thinness

All data were ordinal and non-normally distributed (max Shapiro-Wilk statistic = .93, p = .018); thus, non-parametric statistics were used. When asked whether they felt fatter after being presented with the obese body, there was no significant difference in agreement between the synchronous and asynchronous conditions (z = -1.31, p = .346, r = .15; synchronous median = 2, synchronous IQR = 0–2; asynchronous median = 1, asynchronous IQR = 0–2). There was also no significant difference between the synchronous and asynchronous conditions when examining females only (z = -.459, p = .764, r = .07; synchronous median = 1, synchronous IQR = -1.75–2; asynchronous median = 0, asynchronous IQR = -1.75–2). For males, however, agreement with the statement “I felt fatter than usual” was significantly greater for the synchronous condition (median = 2, IQR = 1–2.75) than for the asynchronous condition (median = 1, IQR = 1–2) (z = -.2.5, p = .039, r = .40). In a direct comparison of the responses between males and females, the agreement did not significantly differ between the sexes for the obese synchronous (z = -1.65, p = .215, r = .26) or obese asynchronous (z = -1.25, p = .364, r = .2) conditions.

When asked whether they felt thinner in the slim body conditions, across the entire sample, there was no significant difference between the synchronous and asynchronous conditions (z = -1.96, p = .142, r = .22; synchronous median = 0, synchronous IQR = -2–1; asynchronous median = -1, asynchronous IQR = -2–0). Furthermore, no significant difference for this question was identified between the synchronous and asynchronous conditions when females (z = -1.54, p = .269, r = .24; synchronous median = 0, synchronous IQR = -3–2; asynchronous median = -.5, asynchronous IQR = -3–1) or males (z = -1.26, p = .364, r = .20; synchronous median = -.5, synchronous IQR = -2–0; asynchronous median = -1, asynchronous IQR = -2–0) were examined independently. In a direct comparison of the responses between males and females, no significant difference was identified for the slim synchronous (z = -.672 p = .622, r = .11) or slim asynchronous (z = -.344, p = .760, r = .05) conditions.

Thus, although the full-body illusion of owning the obese and slim bodies worked well (see above), this was not systematically associated with significant increases in the explicit feelings of being “fatter” or “thinner”. These results are in line with our previous study [44]. Interestingly, males demonstrated a significant effect of synchrony for feelings of fatness in the obese body conditions in contrast to females.

#### Explicit body satisfaction

Across the entire sample, the data from the BISS for all conditions did not significantly differ from a normal distribution (max Shapiro-Wilk statistic = .95, p = .07); thus, parametric tests were used. The data were subsequently entered into a 2x2 mixed effects ANCOVA with body size (slim and obese) and touch (synchronous and asynchronous) as within factors, sex (male and female) as the between factor and the EDE-Q score as a covariate.

There was a significant effect of body size (F(1,37) = 5.66, p = .023, ηp2 = .133) with lower reported body satisfaction following ownership of an obese body (mean = -.85) compared to that following ownership of a slim body (mean = .04). Furthermore, there was a significant size x synchrony x sex interaction F(1,37) = 6.5, p = .015, ηp2 = .149. Moreover, the ANCOVA indicated a significant size x synchrony x EDE-Q score interaction F(1,37) = 4.43, p = .042, ηp2 = .107. All other main effects and interactions did not reach significance (max F(1,37) = 2.8, p = .19, ηp2 = .046).

**Fig 5:**
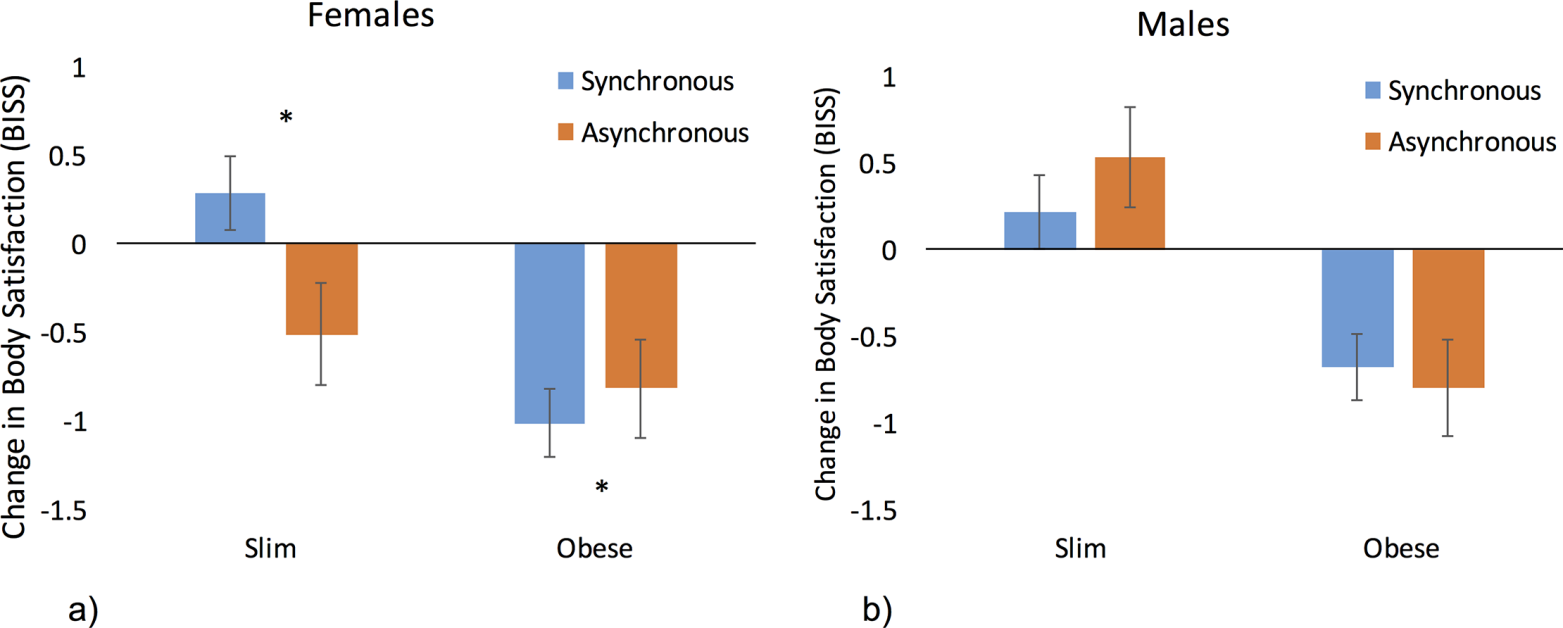
Body size x synchrony x sex interaction. Changes in explicit body satisfaction following synchronous and asynchronous touch applied to both obese and slim bodies for females (a) and males (b). Females demonstrate increased body satisfaction when synchronous touch is applied to a slim body compared to the asynchronous control condition. Relative decreases in body satisfaction were reported for synchronous compared to asynchronous touch applied to an obese body. Males show increases in body satisfaction for a slim body and decreases for an obese body irrespective of touch. Bars represent mean scores and error bars depict standard error. * = p < .05.

To directly test our hypothesis that illusory obesity would result in significantly lower body satisfaction for females, we compared the body satisfaction scores following synchronous and asynchronous touch for both body sizes for females only. The body satisfaction change from baseline was non-normally distributed for the slim synchronous (Shapiro-Wilk statistic = .792, p = .001) and obese asynchronous (Shapiro-Wilk statistic = .790, p = .001) conditions; thus, non-parametric tests were used. For females, body satisfaction was lower following the synchronous (mean = -.58) than asynchronous touch (mean = -.33) of an obese body (z = -1.99, p = .046, r = .31). Our female participants also reported greater body satisfaction when viewing the slim body following the synchronous touch than following the asynchronous touch (synchronous median = .0, asynchronous median = -.33) (z = -2.9, p = .004, r = .46) (Fig 5A). We also compared body satisfaction between body sizes for the synchronous conditions only and importantly determined that for females, body satisfaction was significantly lower following synchronous touch of an obese body (median = -.58) compared to a slim body (median = 0) (z = -2.53, p = .011, r = .31).

We did not expect that body satisfaction would vary as a result of the ownership illusion for male participants in either body size condition. To examine whether the data were in line with this prediction, we compared the body satisfaction scores for the male participants following synchronous and asynchronous conditions for both body sizes and between body sizes for synchronous touch only. All data were normally distributed (min Shapiro-Wilk statistic = .912, p = .069); thus, parametric tests were used. For the males, there were no significant differences in the level of body satisfaction between the synchronous and asynchronous touch for the obese body (synchronous mean = -.65; asynchronous mean = -.8; t(19) = -.61, p = .55, *d* = .14, B = .25) or slim body (synchronous mean = .22; asynchronous mean = .15; (t(19) = .24, p = .81, *d* = .05) (Fig 5B). Thus, in line with our hypothesis, illusory ownership of the obese or slim bodies did not significantly affect body satisfaction in men. Crucially, this was statistically supported for the obese conditions with a Bayes factor of less than 1/3 (B = .25), thus demonstrating support for the null hypothesis. In addition, and expectedly, we noted that body satisfaction was significantly lower following synchronous touch of an obese (mean = -.65) compared to a slim (mean = .22) body (t(19) = -3.12, p = .006, *d* = .70).

We subsequently examined whether body satisfaction for males and females differed from baseline. The body satisfaction change was non-normally distributed for the slim synchronous (Shapiro-Wilk statistic = .792, p = .001) and obese asynchronous (Shapiro-Wilk statistic = .790, p = .001) conditions; thus, non-parametric tests were used for these comparisons, whereas all other comparisons used parametric analysis. For the females, the body satisfaction scores decreased following both the synchronous (t(19) = 3.5, p = .016, *d* = .78) and asynchronous (z = -2.92, p = .016, r = .65) touch of an obese body. Body satisfaction was not significantly different from the baseline following the synchronous touch of a slim body (z = .617, p = .537, r = .14); however, it was significantly reduced following the asynchronous touch of a slim body (t(19) = 2.5, p = .038, *d* = .55). For the males, all data were normally distributed; thus, parametric tests were used. The body satisfaction scores did not differ from baseline for the synchronous (t(19) = 1.34, p = .263, *d* = .30) or asynchronous touch (t(19) = .933, p = .415, *d* = .21) of a slim body. Body satisfaction was significantly lower than baseline following both the synchronous (t(19) = -2.5, p = .024, *d* = .56) and asynchronous (t(19) = -2.95, p = .021, *d* = .66) touch of an obese body.

**Fig 6:**
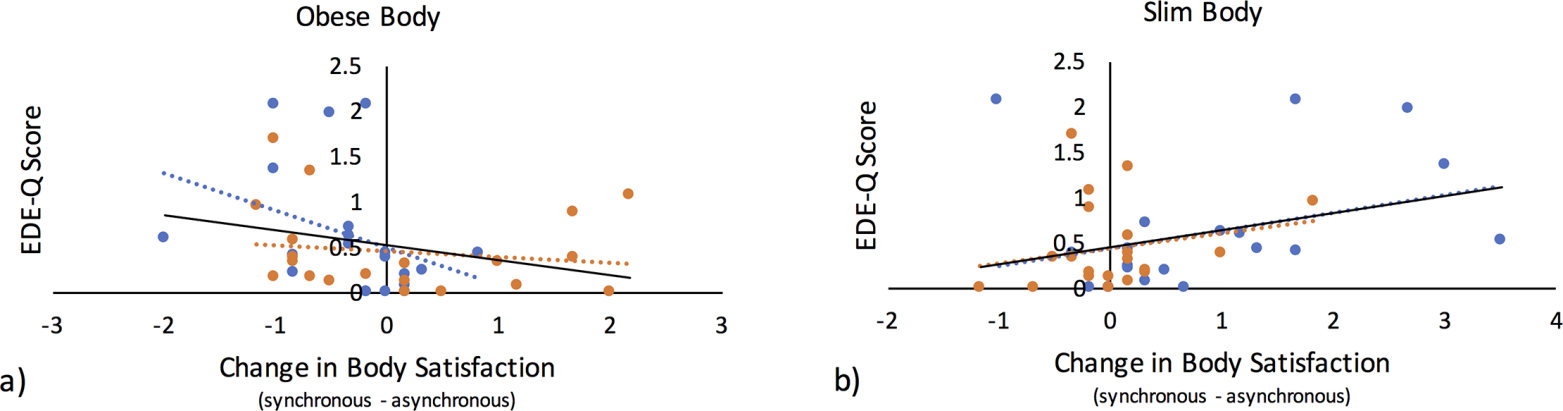
Body size x synchrony x EDE-Q interaction. a) Decreases in explicit body satisfaction were correlated with EDE-Q score in the obese body condition, in which higher cognitive-behavioural eating disorder characteristics were associated with greater reductions in body satisfaction. This relationship survived for females (blue circles) but not for males (orange circles). b) Increases in body satisfaction correlated with EDE-Q score in the obese body condition, in which higher cognitive-behavioural eating disorder characteristics were associated with greater increases in body satisfaction. This relationship did not survive for females (blue circles) or males (orange circles) when examined independently.

To follow up the interaction between size x synchrony x EDE-Q score (see above), we calculated a change in body satisfaction by subtracting the body satisfaction scores following the asynchronous conditions from the scores following the synchronous conditions for each body size separately and subsequently correlated with the EDE-Q scores. Because the data were highly skewed (max Shapiro-Wilk test = .94, p = .027), Spearman’s Rho nonparametric statistics were used. A significant negative correlation was identified for the obese condition (r_s_ = -.385, p = .028), in which higher cognitive-behavioural eating disorder characteristics (EDE-Q) were associated with greater reductions in body satisfaction. Furthermore, a signification positive correlation was identified for the slim condition (r_s_ = .315, p = .047), in which higher cognitive-behavioural eating disorder characteristics were associated with greater increases in body satisfaction (Fig 6). (For correlation results with EDE-Q subscales, refer to S4 Table.)

As an additional follow-up, we examined whether these correlational relationships survived for females and males independently. For females, the relationship survived for the obese body condition (r_s_ = -.537, p = .03), but it did not reach significance for the slim body (r_s_ = .432, p = .056). For males, neither relationship was significant (slim body, r_s_ = .235, p = .319; obese body, r = -.282, p = .319).

#### Correlating feelings of “Fatness/Thinness” and body satisfaction

Spearman’s Rho nonparametric statistics were used as a result of the non-normal distribution of the data (max Shapiro-Wilk statistic = .94, p = .027). There was a trend for a negative correlation between a decrease in body satisfaction and a subjective increase in “fatness” for the obese body (r_s_ = -.34, p = .062); however, this relationship was not identified with the slim body (r_s_ = -.179, p = .27). We also followed up these results by examining males and females independently. For our female participants, there was a significant negative relationship between a decrease in body satisfaction and an increase in subjectively experienced fatness for the obese body (r_s_ = -.61, p = .006), in which the more participants agreed with the statement “my body felt fatter than usual”, the greater decrease in reported body satisfaction in the synchronous compared to asynchronous conditions. There was also a significant positive relationship between a subjective increase in thinness feelings and an increase in body satisfaction when owning the slim body (r_s_ = .53, p = .017), in which the more female participants agreed with the statement “my body felt thinner than usual”, the greater increase in reported body satisfaction in the synchronous compared to asynchronous conditions with the slim body. For males, neither relationship was significant (obese body and feelings of fatness, r_s_ = -.199, p = .703; slim body and feelings of thinness, r_s_ = -.091, p = .703).

### Experiment two

#### Descriptives

There were no significant differences between the male and female participants for any of the demographics measures assessed (age and BMI). However, significant differences were identified for the attitudinal measures. The EDE-Q was higher for the females than the males. Self-esteem was lower for the females than for the males (Table 2).

**Table 2:** Participant demographics experiment two.

Measure	Total	Male	Female	T statistic	P value
Age	26 years (4.4) [18–39]	26 years (4.7) [18–39]	26 years (4.1) [20–35]	.00	1.00
BMI	22.2 (2.1) [18.1–28.6]	22.5 (1.9) [19.6–25.6]	22.01 (2.2) [18.1–28.6]	.88	.38
SE ^c^	22.8 (5) [11–30]	24.5 (4.3) [15–30]	21.1 (5.1) [11–30]	2.7	.009
EDE-Q	.53 (.21-.91) ^a^ [0–2.44]	.31 (.12 - .67) ^a^ [0–1.1]	.80 (.4–1.2) ^a^ [.08–2.4]	3.9^b^	< .001

The table presents the means with standard deviations in parentheses and ranges in square brackets for participant age, body mass index (BMI), self-esteem measured by the Rosenberg Self-Esteem Scale (SE) and cognitive-behavioural eating disorder characteristics measured by the eating-disorder examination questionnaire (EDE-Q) score.

^a^Median and inter quartile range in parentheses

^b^Mann-Whitney U statistic

^c^Data missing for three participants as a result of subjects not completing the questionnaire.

#### Body ownership illusion

The questionnaire data (illusion composite score) for all conditions were ordinal and not normally distributed (max Shapiro-Wilk statistic = .91, p < .001); thus, the data were analysed using non-parametric Wilcoxon signed rank tests. To determine the effect of synchrony of seen and felt touch for each body size condition, we compared the synchronous and asynchronous trials for the obese and slim bodies independently. A significant effect of synchrony was identified with the obese body (z  =   -5.50, p <  .001, r = .50) with higher illusion ratings for synchronous touch (median   =  1.5) than for asynchronous touch (median   =  −1.0). The same was true for the slim body with a higher illusion strength reported in the synchronous condition (z  =   -6.26, p < .001, r = .55, synchronous median =  1.75, asynchronous median   =  −.5) (Fig 4B). However, equivalent tests on the control scores indicated there was no effect of synchrony with the obese (z  = -.378, p  = .705, r = .03) or slim (z   =  -.834, p  = .505, r = .07) bodies. To directly compare the illusion strength for each body size, we compared the illusion scores for the obese and slim bodies following the synchronous trials only. There was no significant difference between the illusion strength in the obese and slim synchronous conditions (z =  -1.87, p  = .103, r = .17). The patterns of the results for the illusion scores were also equivalent for the male and female participants when directly compared and analysed independently (refer to supplementary material S1 Text). Thus, similar to the first experiment, the basic full-body illusion worked as expected.

#### Feelings of Fatness/Thinness

The data were both ordinal and not normally distributed (max Shapiro-Wilk statistic = .92, p < .001); thus, non-parametric tests were used. When asked whether they felt fatter in the obese body conditions, there was a significant difference between the synchronous and asynchronous conditions across the entire sample (z = -2.66, p = .023, r = .24) with synchronous (median = 2) having stronger agreement than asynchronous (median = 1). However, when females and males were independently examined, no significant effect of synchrony was identified for the females (z = -1.65, p = .15, r = .21) or the males (z = -2.0, p = .09, r = .25). In a direct comparison of the responses between males and females, agreement on the statement of feeling fatter in the obese conditions did not indicate significant differences for the obese synchronous (z = -1.1, p = .349, r = .13) or obese asynchronous (z = -1.77, p = .125, r = .22) conditions.

When asked whether they felt thinner during the slim body conditions, there was no significant difference between the synchronous and asynchronous conditions across the entire sample (z = -1.07, p = .353, r = .09). Similarly, no significant differences in the subjective thinness ratings were identified between the synchronous and asynchronous trials for the females (z = -1.43, p = .216, r = .18) or males (z = -.10 p = .955, r = .01) independently.

A direct comparison of males and females indicated that the females demonstrated significantly greater agreement for feelings of thinness in both the slim synchronous (male median = -1, female median = +1, z = -3.08, p = .011, r = .38) and slim asynchronous (male median = -1, female median = 0, z = -2.38, p = .044, r = .30) conditions. Refer to the S2 Table for the scores on the other questionnaire items.

#### Implicit body satisfaction (IAT)

The d scores in each of the conditions were subtracted from the baseline scores and subsequently entered into a 2x2 mixed ANCOVA with the factors body size and synchrony, the between variable as the sex of the participant and a covariate of the EDE-Q score. There was a significant main effect of Synchrony (F(1,61) = 7.71, p = .007, ηp2 = .11) with synchronous being higher (mean = -21.17) than asynchronous (mean = -39.23) (although both were negative). There was also a significant Synchrony x EDE-Q interaction (F(1,61) = 6.26, p = .015, ηp2 = .093) (refer to follow-up analysis below). All other main effects and interactions did not reach significance (max F(1,61) = 1.02, p = .316, ηp2 = .017).

To directly test our hypothesis that illusory obesity would result in significantly lower implicit body satisfaction (i.e., a lower d score) for females, we compared the d scores following the synchronous vs. asynchronous touch for both the slim and obese body sizes for females. We subsequently compared the IAT scores between the body sizes for the synchronous touch trials only. The data for the females only were non-normally distributed for the slim synchronous (Shapiro-Wilk statistic = .920, p = .021) and the slim asynchronous (Shapiro-Wilk statistic = .913, p = .014) conditions; thus, non-parametric tests were used for analyses with these variables. There was no significant difference between the d scores from our female participants for the synchronous and asynchronous touch for a slim body (z = -.561, p = .575, r = .07) or an obese body (t(31) = 1.47, p = .152, *d* = .26). However, the d scores were significantly lower following the synchronous touch of an obese (median = -.57) compared to a slim (median = -.20) body (z = -2.26, p = .024, r = .28).

We subsequently examined the same effects for our male participants only. All data were normally distributed (min Shapiro-Wilk statistic = .958, p = .238); thus, parametric tests were used. There were no significant differences between the d scores from our male participants for the synchronous and asynchronous touch for a slim body (t(31) = 1.5, p = .144, *d* = .27) or an obese body (t(31) = 1.03, p = .307, *d* = .18). Furthermore, the d scores were not significantly different following the synchronous touch of an obese compared to a slim body (t(31) = .387, p = .701, *d* = .07). Therefore, despite the main effect of synchrony, differences between synchronous and asynchronous touch were not identified for either body size when examined individually for males and females.

We subsequently examined whether the d scores for males and females differed from the baseline by conducting one sample tests. The data were non-normally distributed for the slim synchronous and the slim asynchronous for the female (see above) conditions; thus, non-parametric tests were used for these comparisons, whereas all other comparisons used parametric analysis. For the females, the scores were not significantly different from baseline following synchronous (t(19) = 1.93, p = .461, *d* = .34) or asynchronous (t(19) = 1.14, p = .461, *d* = .20) touch for an obese body. The d scores were also not significantly different from baseline following synchronous (z = -.505, p = .818, r = .06) or asynchronous (z = -1.5, p = .461, r = .19) touch of a slim body. For the males, the d scores also did not differ from baseline for any of the conditions (obese synchronous t(19) = -.15, p = .912, *d* = .03; obese asynchronous touch t(19) = 1.19, p = .461, *d* = .21; slim synchronous t(19) = -.11, p = .912, *d* = .02; slim asynchronous t(19) = -1.1, p = .461, *d* = .19).

**Fig 7:**
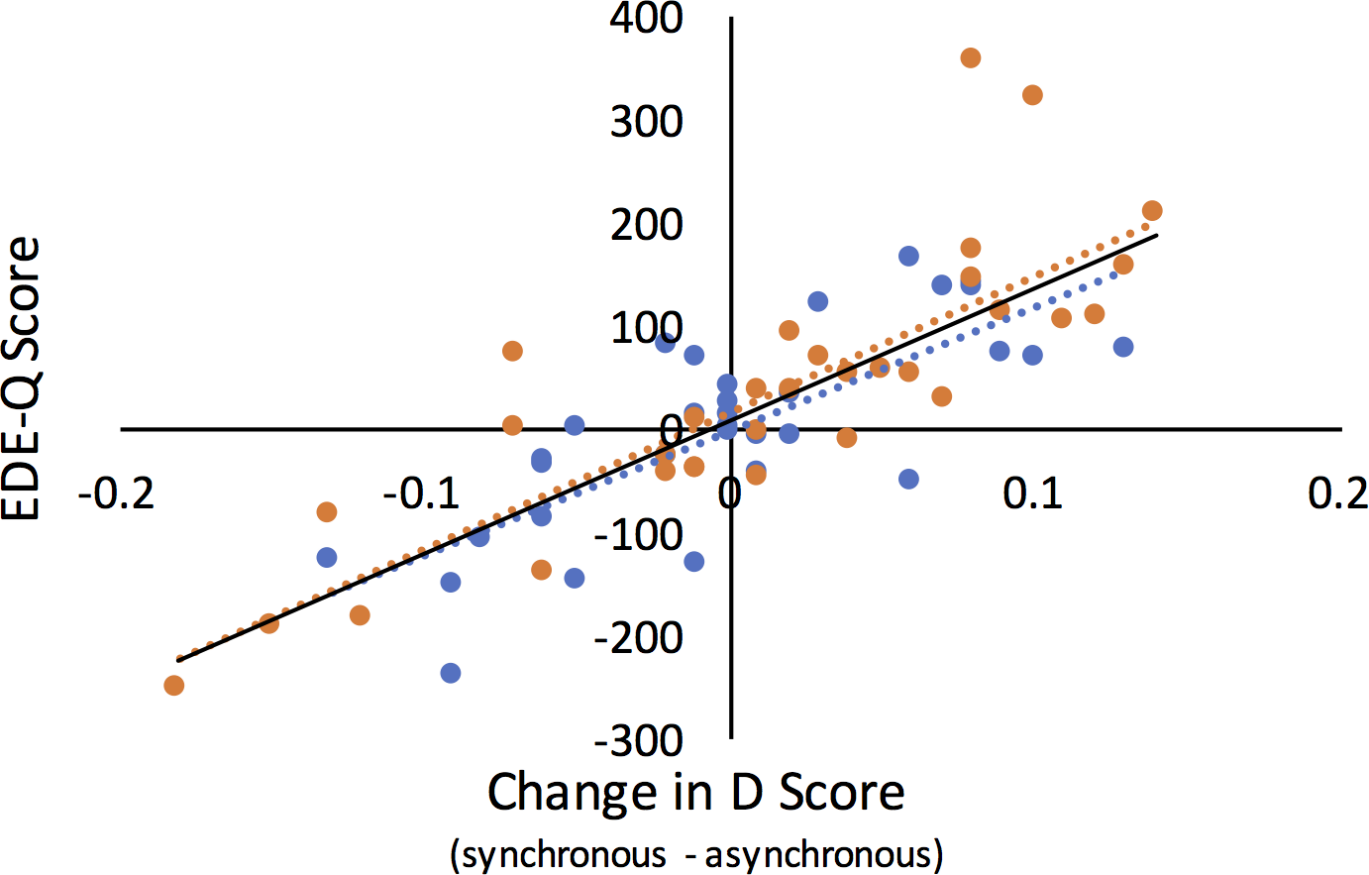
Synchrony x EDE-Q interaction. Increases in implicit body satisfaction (d score) correlated with EDE-Q score across body size conditions, in which relative increases in d scores corresponded with lower non-clinical cognitive-behavioural eating disorder characteristics (EDE-Q score). Female participants are represented by blue circles, and male participants are represented by orange circles. EDE-Q = eating disorder examination questionnaire.

To follow up the Synchrony x EDE-Q interaction, the IAT scores following asynchronous touch were subtracted from the scores following synchronous touch across body size and were subsequently correlated with the EDE-Q scores. Because the EDE-Q data were non-normally distributed (Shapiro-Wilk statistic = .87, p < .001), Spearman’s Rho nonparametric statistics were used. A significant negative correlation was identified (r_s_ = -.341, p = .006), in which greater increases in the IAT score for synchronous touch relative to asynchronous touch were associated with lower EDE-Q scores (Fig 7). (For correlation results with EDE-Q subscales, refer to S5 Table).

Because of the hypothesised difference in the effects of the illusion between males and females, as well as the identified significant difference between males and females in the EDE-Q score (Table 2), this interaction was further followed up by examining the relationship between the change in d score and EDE-Q score for males and females independently. For both females and males, this correlation approached significance (females: r_s_ = -.318, p = .076, males: r_s_ = -.313, p = .08).

## Discussion

The current study investigated explicit and implicit measures of body satisfaction as a result of illusory ownership over slim and obese bodies in males and females. There were three main findings. First, and in support of our hypothesis, we found that ownership of a different body size had a direct effect on explicit body satisfaction in females but not males. Specifically, ownership of the obese body in females led to reductions in body satisfaction as reported on the BISS scale. This finding extends our previous findings obtained during fMRI [45] to the more controlled experimental design and laboratory environment of the current study. Second, and in contrast to our hypothesis, the implicit measures of body attitudes/satisfaction were not systematically influenced by the size of the illusory owned body for either sex. Unexpectedly, there was a significant effect of synchrony of touch across obese and slim bodies and across both sexes; thus, the body ownership illusion seemed to increase implicit associations between words related to self and attractiveness regardless of the size of the body or the sex of the participant. Third, and interestingly from a clinical perspective, we determined that changes in body satisfaction for both implicit and explicit measures were related to non-clinical cognitive-behavioural eating disorder characteristics. In line with our hypothesis, females at risk of developing eating disorders, i.e., individuals with relatively high EDE-Q scores, reported greater body dissatisfaction in response to the illusory obesity. This finding supports the notion that this group has a less stable affective representation of their body and that they therefore respond with greater body dissatisfaction to perceived increases in body size. Unexpectedly, we also determined that individuals (both males and females) with high EDE-Q scores showed significantly smaller increases in implicit body satisfaction when experiencing the body ownership illusion (irrespectively across body size), again supporting an altered affective body representation in this group. Taken together, our findings illustrate how full-body ownership illusions may be used as an experimental tool to investigate how dynamic changes in body satisfaction are triggered by perceived changes in body size, particularly in individuals with high non-clinical cognitive-behavioural eating disorder characteristics.

### Explicit body satisfaction

The present results for explicit body satisfaction extend our previous studies [44,45] in several important ways. First, we demonstrated that the illusion of owning an obese body produced significant reductions in body satisfaction in women using a validated questionnaire, thus extending our previous observation that these reductions could be identified on single item ratings [45]. Moreover, in the present study, we used real human models of obese and slim strangers rather than a mannequin that was digitally manipulated to look larger or thinner as in our previous studies [44]. In one previous study, the images of the mannequin were linearly stretched or compressed, making the mannequin look 15% larger or 15% thinner. However, this manipulation is unrealistic and does not capture the look of a real ‘socially undesirable’ obese body as the mannequin retained a flat stomach during the digital manipulations. This difference may explain why we identified significant decreases in body satisfaction with owning the obese model in the current experiment and our previous fMRI experiment [45], whereas owning the large mannequin [44] did not decrease body satisfaction. Thus, ecologically valid stimuli of real strangers’ obese bodies are likely preferable over mannequins in body satisfaction studies.

From a cognitive/psychological perspective, the current results that relate explicit body satisfaction to illusory obesity (and illusory slimness) are important because they further support a direct link between perceived body size and body satisfaction in women, thus contributing to previous findings of interconnectivity between perceptual and affective body representations in the brain [45]. Therefore, illusory ownership over an obese body results in lower reported body satisfaction for female participants. This finding suggests that perceived (regardless of whether it is real) weight gain may have a negative impact on the explicit emotional well-being of women. This finding may also have clinical relevance with low body satisfaction implicated in the development and maintenance of eating disorders [1]. Despite increasing levels of obesity globally [66], in today’s modern society, the pressures on physical appearance of the body, particularly for women, remain high with a slim physique being the most socially desirable. A direct link between body perception and body affection in the context of this increasing disparity between unattainable social ideals and the average woman’s body size may therefore be a fundamental factor that contributes to recent reported increases in admissions for eating disorders [67–70]. The observation of this relationship with females and not males is also in line with previous findings that women have stronger negative responses to increases in body size [5,16], greater negative responses to illusory obesity during fMRI [45], and a greater incidence of eating disorders in the clinic [70].

Body satisfaction for males is likely to be more complex than simple increases in body size as it is also thought to be strongly influenced by muscle mass [71]. However in this study, the obese male body was both large and not overtly muscular. In line with our hypothesis, the illusion of owning the obese body did not lead to a specific reduction in body satisfaction in the male group, which supports previous findings of minimal negative effects of real and perceived obesity in men [e.g. 6, 45]. However, unexpectedly for males, body satisfaction was reduced to an equivalent degree in both synchronous and asynchronous obese conditions. Why body satisfaction was reduced in both conditions in this way in men is unclear. It may be that viewing the body from a first-person perspective is sufficient to elicit weak, non-explicit forms of ownership (which we refer to here as residual ownership) over the obese body, given that the only body we view normally from this perspective is our own. It is notable that even if participants tended to deny explicit feelings of ownership of the bodies in view during asynchronous touch, the median illusion scores were not strongly negative (Fig 4). Moreover, a further analysis that examined the differences in ownership for the asynchronous trials only indicated there was no significant difference in illusion extinction as a result of asynchronous touch (agreement to the illusion relevant questionnaire items in the asynchronous conditions) between the sexes for either body size (slim z = -.1.27, p = .203, r = .2; obese z = -1.19, p = .236, r = .19).

Our female participants also demonstrated a reduction in body satisfaction in the obese asynchronous condition, and this reduction was not significantly different to the obese asynchronous condition for males (z = -.298, p = .766, r = .05; however, as stated in the results section, body satisfaction was significantly further reduced for females following synchronous touch compared to asynchronous touch). We also noted that there was no significant difference between synchronous and asynchronous touch for the statement “my body felt fatter than usual” for either sex (however, this difference was significant across both sexes in experiment two with more participants). Thus, with the obese body, both males and females agreed with feeling fatter for both the synchronous and asynchronous conditions. This finding may suggest residual ownership in the asynchronous conditions that influences body satisfaction and feelings of fatness, while not eliciting explicit feelings of ownership. However, why this should vary between males and females is unclear. Thus, future studies should further explore the influence of visual perspective on body ownership to address these questions.

Another interesting finding from the current study was obtained during the slim body conditions. Here, for women, we identified relatively higher explicit body satisfaction during synchronous touch than asynchronous touch. This finding may be a result of an increased body satisfaction as a result of ownership over a slimmer body. Although our previous experiment using the same stimuli during fMRI did not identify an increase in body satisfaction following illusory ownership of the slim body [45], increases in body satisfaction were recorded following ownership over a mannequin body digitally adjusted to be 15% slimmer than the participant [44]. These findings suggest that ownership over slimmer bodies may increase body satisfaction in some cases. However, one sample tests indicated this significant effect of synchrony to be driven more by a reduction of body satisfaction in the asynchronous condition compared to baseline, rather than an increase in body satisfaction in the synchronous condition. It has been well documented that viewing slim (ideal) bodies of other individuals may reduce body satisfaction in females [72–75]. This is thought to be a result of the observer making comparisons between the viewed ideal bodies and their own body. Therefore, body comparison may explain the current observed reduction in body satisfaction in females during the slim asynchronous condition. Asynchronous touch indicates that the observed body does not feel like it is the participant’s own body (supported by the relatively low ownership scores in these conditions); thus, comparisons may be made between the viewed body and the actual body, the former of which is slimmer, leading to a reduction in body satisfaction [14]. Similarly, it has also been suggested that viewing overweight/obese bodies of other individuals may increase body satisfaction [75], presumably as a result of similar mechanisms. However, the current study identified a decrease in body satisfaction during asynchronous touch of an obese body (see above), the opposite effect that you would expect from body comparison. Therefore, neither an explanation of residual ownership driven purely by a first-person perspective (see above), nor that of body comparison between the asynchronously touched (not owned) body and the actual body can successfully account for the observations from our female participants in the asynchronous conditions for both body sizes. Thus, these seemingly contradictory results may suggest that we process information regarding other individuals’ bodies differently depending on body size.

The relationship between body satisfaction change and ownership over a slim body deserves further consideration. Although our samples are often far closer to the slim body for actual body size than the obese body, BMI and difference between participant and model BMIs have not previously been shown to account for the changes in body satisfaction [45]. However, the examination of males and females independently in the current experiment indicated a significant relationship between increases in body satisfaction during ownership of a slim body and increases in participants’ BMI for females (refer to supplementary material). These results are important because they support the assertion that closeness in size between our participants’ bodies and that of the slim model prevents changes in body satisfaction. Furthermore, it suggests that BMI is a key feature in driving body satisfaction over illusory owned slim bodies. However, the fact that this relationship does not hold for an obese body suggests that the BMI of normal participants, as used in the present study, is not as important for reported feelings of body dissatisfaction during illusory obesity. Moreover, the BMI measurement does not take into account relative amounts of muscle and fat distributions. Therefore, BMI may not be an adequate index of body size, at least not for every individual. Future research may consider incorporating other measurements of body size, particularly when assessing males, for which muscle mass may be as an important contributor to body satisfaction as fat [13,57].

A recent study indicated that exposure to overweight bodies viewed from a third person perspective led to larger judgements of own body size in anorexic patients, whereas similar effects were not identified following exposure to slim bodies or when healthy controls observed either body type [76]. The authors suggested that this increase in self body size judgements was a result of perceptual aftereffects, in which exposure to larger bodies causes the figure in the body size estimation task to appear thinner relative to an internal representation of the self, hence the increase in body distortion in this condition. There is evidence that perceptual aftereffects of this nature only occur for large and not slim bodies [77], thus explaining the asymmetry in body judgements for the different body sizes. However, we suggest that perceptual aftereffects are unlikely to be the cause of the current results, given that exposure to the different body sizes was substantially less than previous studies (30 seconds in the current study vs. 8 minutes in [76]) and ratings of body satisfaction were made in the absence of visual feedback of their own body or a reference body. This second aspect is crucial given that without a visual reference body presented, perceptual aftereffects would only be able to be compared to the internal representation, which was claimed to be unchanged [76]. If this internal representation is unaffected, perceptual aftereffects should, if anything, result in an increase in body satisfaction as the internal self-representation would appear thinner relative to the larger body in view. Furthermore, in the previous study, the authors did not identify these effects in healthy individuals, even after 8 minutes of exposure [76]. However, the current study identified significant effects on body satisfaction in healthy individuals following only 30 s of exposure to illusory ownership of an obese body, which indicates that the two studies uncover different mechanisms. A key difference between the current study and [76] is that during both present illusion and non-illusion conditions, our participants viewed the bodies from a first-person perspective, a view of the body more readily associated with the self and that produces significantly stronger ownership perceptions than bodies viewed from a third-person perspective [27,78]. Thus, it may be that the brain processes information regarding the body differently when presented from different visual perspectives. Although a previous study with obese individuals indicated that body size estimates were stable irrespective of the visual angle from which a body was presented, images from the first-person perspective were not included [79]. Therefore, future studies should examine the role of visual perspective, particularly regarding first and third person views, in relation to body size processing, to understand how this may influence how we feel about our own and other individuals’ bodies.

A major finding of our results from a clinical perspective is the significant relationship between non-clinical eating disorder characteristics and explicit body satisfaction change for females. This finding suggests a link between the present experimentally evoked changes in body satisfaction and non-clinical eating disorder traits/behaviours in women. This finding is in line with previous studies that indicate individuals with eating disorders or high non-clinical eating disorder characteristics are more susceptible to emotional changes, particularly regarding the body [44,80]. However, previous research has suggested that individuals who suffer from eating disorders [19,81] and healthy individuals with high non-clinical eating disorder characteristics [82] have a stronger experience of multisensory illusions involving the hand (rubber hand illusion), in which increases in emotional response to owning different sized bodies may only be a consequence of a stronger illusion in these participants. However, a follow-up analysis on the entire sample (combining scores from both sexes in experiments one and two to increase power) failed to identify a significant relationship between illusion strength and non-clinical eating disorder characteristics (Spearman’s Rho correlation EDE-Q with illusion score [synchronous minus asynchronous]: r_s_ = -.027, p = .735). Thus, individuals with high eating disorder cognitive behavioural characteristics did not experience a stronger full-body ownership illusion in the present study; therefore, body ownership *per se* cannot explain why females with high non-clinical eating disorder characteristics reported greater body dissatisfaction during illusory obesity. Considering that this observation is in apparent conflict with the previous result from the rubber hand illusion discussed above, we speculate that it may be that increased perceptual malleability of the body is only apparent with non-emotionally salient body parts, such as the hand. Illusion strength has been shown to be equivalent in anorexia patients and controls for a similar full body illusion using an avatar [83], and previous findings that link rubber hand illusion susceptibility to non-clinical eating disorder characteristics in healthy individuals only identified these relationships with the left and not the right hand [82], which suggests that these findings may be body part specific. Finally, our current results show that increases in subjectively felt fatness (agreement with “my body felt fatter than usual”) correlate with body satisfaction changes in females; thus, the greater the subjective fatness induced by the illusion of owning the obese body, the greater the reduction in body satisfaction. However, an additional correlation analysis indicates that even this specific aspect of the illusion does no correlate with the EDE-Q (Spearman’s Rho correlation EDE-Q with agreement with “my body felt fatter than usual”: r_s_ = -.012, p = .91). Thus, our findings suggest that otherwise healthy females with relatively high (within the current sample) non-clinical eating disorder characteristics have a stronger change in body satisfaction during the illusion of owning the obese body. It has been widely reported that individuals with eating disorders, particularly anorexia nervosa, have an inaccurate perception of their own body, specifically feeling they are fatter than they are in reality [18–22], and this overestimation of body size has been linked to poor prognosis [21,24]. Our current findings suggest that individuals with higher non-clinical eating disorder characteristics experience an exaggerated negative response to perceived increases in weight. If this is also combined with a distorted experience of their actual body size, thought to be present in clinical eating disorder samples, it may exacerbate negative feelings regarding their body. Therefore, the link between affective and perceptual body representations may provide a mechanism that underlies worse clinical outcomes in patients with more inaccurate bodily experiences [21,24]. Furthermore, as this relationship was identified in a healthy non-clinical sample, this heightened emotional (body dissatisfaction) response to increases in body size may also be important for the development of eating disorders rather than only being a consequence of the physical effects of the disease. However, further studies must examine this relationship in eating disorder patients to determine whether the patterns identified here in healthy samples are mirrored in the clinic.

Obese individuals have also been reported to overestimate the dimensions of their own body parts, both in terms of visual-mental representation and when judging the distance between two tactile points on the skin [84]. Moreover, obese individuals are thought to overestimate real world distances compared to slimmer individuals, which may contribute to health related behaviours, such as choosing whether to drive or walk to work [85,86]. Therefore, the current findings regarding the malleability of body perception and a link between body perception and body satisfaction may also be important for future research related to the mechanisms that underlie the development of obesity and the effectiveness of incorporating body illusions into weight-loss programmes. A case study that implemented a full body ownership illusion using a slim virtual reality avatar with a super-super obese (BMI > 60) female participant indicated improvements in anxiety associated with her clinical condition, motivation with treatment and body size estimations [87].

Exactly why female participants feel more dissatisfied with their body during perceived obesity and why this is more pronounced in individuals with relatively high non-clinical eating disorder characteristics is not clear from the current results. Disgust, fear of fatness and anti-fat attitudes have been linked to concerns regarding physical appearance [88], as well as aesthetic judgements [89]. These components are potential candidates to mediate the strength of the relationship between perceptual and affective body representations. Future studies should specifically aim to gain a clearer overall understanding of the mechanisms that drive the negative body satisfaction effect of perceived changes in body size as this may be important for the development of prevention strategies for body dissatisfaction and ultimately eating disorders. In our current study, we measured aesthetic judgements in the form of attractiveness ratings (refer to supplementary S1 Text). Unsurprisingly, for both males and females, obese bodies were considered less attractive than slim bodies; however, the attractiveness ratings were not modulated by the experience of the illusion and were not correlated with the body satisfaction ratings. Therefore, although aesthetic preferences and attractiveness are likely to be important for body satisfaction, attractiveness in isolation cannot explain the current findings.

The finding that the relationship between body satisfaction and body perception is stronger for females than males is in line with previous studies, suggesting that women have more dissatisfaction with their bodies [6–8], greater negative effects of increases in body size [6,16] and a greater incidence of eating disorders in the clinic [90]. However, recent research focused on men and male body dissatisfaction suggests that it may be more complex than only related to the size of the body, with muscle tone and size also being important [72]. Although we selected our male slim model because of the muscular physique (in line with social ideals), the current experiment was primarily designed to assess body size (e.g., we did not measure muscle mass in our current participants or assess subjective changes in muscle mass); thus, it may not be optimal to tap into male body concerns. Similarly, although the EDE-Q shows similar internal consistency and reliability in males and females [59,61], the scale may be more relevant to female body concerns and may not capture all clinically relevant thoughts and behaviours in men [91,92]. Scores on the EDE-Q have been reported to be significantly lower for males than females in both clinical [93,94] and community [95–97] samples; however, as studies on males remain relatively scarce, particularly in the clinic, it is unclear whether this finding reflects a true difference in severity or an inadequacy of the EDE-Q to fully capture male eating disorder symptomology. Future studies should further examine EDE-Q clinical norms in both sexes, as well as incorporate measures and paradigms that specifically target male body satisfaction.

### Implicit body satisfaction

The implicit body satisfaction results from experiment two indicate unexpected findings. We had predicted that the results with the IAT would mirror the results obtained with the explicit measure (BISS); however, this was not the case. We only identified a positive effect of the synchrony of touch and not size of the body being owned in the illusion. Thus, the results from the IAT suggest that the full-body ownership illusion increased the implicit association between self and attractive words irrespective of whether the body in view was obese or slim. We also noted that for all experimental conditions, there was a relative decrease in the IAT d score compared to the baseline, which is likely a result of practice effects increasing performance on the incompatible trials as the only block not to be counterbalanced was baseline—always being completed first. It may be that the experience of owning a different sized body for only 30 s may not be sufficient to significantly influence implicit conceptual associations held towards oneself, or perhaps the repetition of the IAT in the current within participants’ experimental design dilutes this relationship; alternatively, our study might be under powered. However, 30 s illusions and repeated measures of body satisfaction have been shown to elicit changes in the brain [45], as well as explicit changes in body satisfaction (experiment one) with smaller samples. Alternatively, the current IAT, which measures associations between self and attractive in terms of word categories at a conceptual-sematic level, may not capture the subjective changes in body satisfaction that involve affective body representation and that are registered by the BISS questionnaire. However, another potential explanation for the current findings is that implicit body satisfaction is genuinely enhanced by experiencing a full-body illusion *per se*, irrespective of the size of the owned body. Multisensory illusions similar to the one described in this study have been shown to be enjoyable for participants [98], and as happiness is positively associated with body satisfaction [99], this general positive affect might increase implicit positive associations concerning our physical appearance. However, we did not explicitly measure the enjoyment of the illusion during the experiment or the overall mood of the participant; thus, this interpretation remains speculative. However, our data indicated a decrease in explicit body satisfaction during the obese conditions, which does not support a general positive influence on affect in our illusion paradigm.

Another speculative explanation for our IAT results is that implicit associations towards the body may be influenced by interoceptive stimuli, e.g., the experience of affective touch. The velocity of touch applied to the observed and felt bodies in these illusions (10 cm/s) falls within the optimal velocity range to activate C fibres associated with affective touch [100–102]. These C fibres, which are thought to be responsible for feelings of pleasantness when the hairy skin is touched gently (affective touch) at a velocity between 1 and 10 cm/s, project to the posterior insular cortex [100,101], which, in turn, projects to the anterior insular cortex, the latter of which is a region of the brain thought to be the interface between emotional states and the body [103,104], responsible for monitoring bodily signals, producing a subjective representation of our body [105,106] and linked to illusory obesity [45]. In addition, the anterior insular cortex is implicated in implicit emotional processing [107]. Therefore, we speculate that interoceptive signals, specifically affective touch as administered in the present paradigm, may play a role in implicit feelings towards our own body. However, why these signals would be stronger in the current synchronous trials than in the asynchronous trials with an identical velocity of touches is not entirely clear; although a recent study on the rubber hand illusion reported significantly greater feelings of pleasantness after synchronous stroking compared to asynchronous stroking [108]. The present experiment was not specifically designed to investigate affective touch; thus, the potential relationships between affective touch and perceptual and affective body representations should be examined in future studies.

We should stress here that from a clinical perspective, the present IAT results are potentially very interesting. The divergence in the IAT d score between synchronous and asynchronous conditions was correlated with cognitive-behavioural eating disorder characteristics and thus supports the assertion that implicit emotional changes as a result of these body illusions are linked to eating disorder traits and behaviours in a healthy population. Although this relationship was similar for males and females, the EDE-Q might not be adequate to capture all aspects of body concern and behaviours for males (refer to previous discussion), and although most of the words used for the IAT were gender neutral, ‘beautiful’ is arguably more feminine, which may have influenced the results (S3 Table). Individuals with eating disorders are thought to have a less stable perceptual representation of the body [19,81], and both eating disorder patients and healthy individuals with relatively high eating disorder characteristics are thought to have a less stable affective representation of the body [44,45]. The current results expand from these previous findings, which suggest that instability of affective body representations extends to implicit measures. Although the underlying mechanism for these implicit emotional changes remains unclear, these new findings support the validity of using multisensory body ownership illusions for further research in eating disorders. Furthermore, because these illusions have been shown to influence both explicit and implicit feelings towards the body, as well as body perception, the current results also support the possibility of using these methods in a therapeutic setting.

### Conclusions

In the current experiment one, we identified further evidence for a direct link between perception of the body and explicit emotional experience of the body (body satisfaction) in our female sample. These findings support previous studies that suggest changes in perceived body size may directly modulate body satisfaction, particularly in women [44,45]. In terms of implicit feelings towards the body examined in experiment two, we identified relatively greater associations between self and attractive stimuli following synchronous touch regardless of the size of the body in the illusion across the entire sample. This finding was unexpected; however, it may be important for informing future research examining links between overall affect, emotional experience of the body, and multisensory body perception. Importantly, both implicit and explicit emotional changes were related to cognitive-behavioural eating disorder characteristics. These findings support the idea that individuals with more eating disorder thoughts and behaviours have a less stable affective representation of the body and therefore respond with greater subjective body dissatisfaction to illusory (and real) increases in body size. Therefore, our results may have important clinical implications for informing the development of future treatment and prevention strategies for eating disorders. Moreover, the current study, within the wider context of previous research related to body perception and disordered eating [18–20,44,81], may suggest multisensory body illusions as a useful clinical tool for future treatments as they influence both bodily perception [19] and bodily emotion [44,45].

## Supporting information

S1 Table Eating disorder examination questionnaire scores. Global and subscale medians (IQR) for the eating disorder examination questionnaire in experiments one and two.
(DOCX)

S2 Table Illusion questionnaire. Medians (IQR) of Illusion Questionnaire items for experiments one and two.
(DOCX)

S3 Table Implicit association task. Words used for the Implicit Association Task in experiment two.
(DOCX)

S4 Table Additional correlations Exp 1. Spearman’s Rho correlations for additional variables in experiment one.
(DOCX)

S5 Table Additional correlations. Spearman’s Rho and Pearson correlations for additional variables in experiment two: Sync vs Async = change in IAT d score for synchronous minus asynchronous trails (across body sizes).
(DOCX)

S1 Text Supplementary results. Additional results for questionnaire items in experiments one and two.
(DOCX)

S1 Data Spreadsheet for main results. Spreadsheets of main results for experiments one and two.
(XLSX)
