# Supplementary material for: Implicit and explicit changes in body satisfaction evoked by body size illusions: Implications for eating disorder vulnerability in women
Source: PLoS One. 2018 Jun 21;13(6):e0199426. doi: 10.1371/journal.pone.0199426 (PMC6013093; doi:10.1371/journal.pone.0199426)
Supplement: S1 Table — Global and subscale medians (IQR) for the eating disorder examination questionnaire in experiments one and two. (DOCX) [file pone.0199426.s001.docx]

**S1 Table. Eating disorder examination questionnaire scores.** Global and subscale medians (IQR) for the eating disorder examination questionnaire in experiments one and two.

|  |  | Global | Restraint | Eating Concern | Weight concern | Shape concern |
| --- | --- | --- | --- | --- | --- | --- |
| Experiment one | Male | .32 ( .11 - .79) | 0 (0 - 1) | 0 (0 - .2) | .4 (0 - .6) | .63 (.25 - .88) |
|  | Female | .46 (.11 - .68) | 0 (0 - .35) | .2 (0 - .4) | .4 (0 -1.35) | .63 (.19 -1.34) |
|  | Total | .35 (.11 - .68) | 0 (0 - .4) | 0 (0 - .2) | .4 (0 - .95) | .63 (.25 - 1.13) |
| Experiment two | Male | .31 (.12 - .67) | .1 (0 - .4) | 0 (0 - .2) | .2 (0 - .6) | .5 (.25 - .97) |
|  | Female | .8 (.4 -1.22) | .7 (.2 - 1.20) | .2 (0 - .55) | .9 (.4 -1.75) | 1.5 (.78 -1.84) |
|  | Total | .53 (.21 - .91) | .2 (0 - .8) | .1 (0 - .4) | .4 (.2 - 1.15) | .88 (.38 - 1.63) |
