# Supplementary material for: Implicit and explicit changes in body satisfaction evoked by body size illusions: Implications for eating disorder vulnerability in women
Source: PLoS One. 2018 Jun 21;13(6):e0199426. doi: 10.1371/journal.pone.0199426 (PMC6013093; doi:10.1371/journal.pone.0199426)
Supplement: S2 Table — Medians (IQR) of Illusion Questionnaire items for experiments one and two. (DOCX) [file pone.0199426.s002.docx]

**S2 Table. Illusion Questionnaire.** Medians (IQR) of Illusion Questionnaire items for experiments one and two.

|  | Experiment one | | | | Experiment two | | | |
| --- | --- | --- | --- | --- | --- | --- | --- | --- |
| Question | Slim Sync | Slim Async | Obese Sync | Obese Async | Slim Sync | Slim Async | Obese Sync | Obese Async |
| ^a^ I seemed to feel the touch given to the body in the image | 2 (2 – 3) | -.5 (-2 – 2) | 2 (1 – 3) | -1 (-2 – 1) | 2 (2 – 3) | -1 (-3 – 1) | 2 (1.25 – 3) | -1 (-2.75 – 1) |
| ^a^ It felt like the body in the image was my body | 2 (1 – 2) | 1 (-2 – 2) | 1 (-1 – 2) | -1 (-2.5 – 1) | 1 (-.75 – 2) | -1 (-2 – 1) | 1 (-1 – 2) | -2 (-3 – 1) |
| ^b^It felt like I had two bodies | -2 (-3 – 0) | -1 (-3 – 1) | -2 (-3 – 0) | -1 (-3 1.5) | -1.5 (-3 – 0) | -1 (-3 – 1) | -1 (-2 – 1) | -1 (-3 – 1) |
| ^b^It felt like my body was two-dimensional | 0 (-3 – 1) | -1 (-3 – 0) | 1 (-3 – 0) | -2 (-3 – .75) | -2 (-3 – 0) | -1 (-3 – 0) | -1 (-3 – 0) | -1 (-3 – 0) |
| My body felt fatter than usual | -2 (-3 – -1) | -2 (-3 – 0) | 2 (0 – 2) | 1 (0 – 2) | -2 (-3 – -1) | -2 (-3 – 0) | 2 (1 – 3) | 1 (-1.75 – 2) |
| My body felt thinner than usual | 0 (-2 – 1) | -1 (-2 - .5) | -3 (-3 – -1.5) | -2 (-3 – -1) | 0 (-2 – 1) | 0 (-2 – 1) | -3 (-3 – -2) | -2 (-3 – 0) |
| The body in the image was attractive | 1 (.5 – 2) | 1 (0 – 2) | -2 (-3 – -1) | -2 (-3 – -1) | 1 (0 – 2) | 1 (0 – 2) | -2 (-3 - -1) | -2 (-2.75 – -1) |

^a^ = Illusion questions, ^b^= control questions
