# Supplementary material for: Implicit and explicit changes in body satisfaction evoked by body size illusions: Implications for eating disorder vulnerability in women
Source: PLoS One. 2018 Jun 21;13(6):e0199426. doi: 10.1371/journal.pone.0199426 (PMC6013093; doi:10.1371/journal.pone.0199426)
Supplement: S3 Table — Words used for the Implicit Association Task in experiment two. (DOCX) [file pone.0199426.s003.docx]

**S3 Table. Implicit Association Task.** Words used for the Implicit Association Task in Experiment two

| **Self** | **Other** | **Attractive** | **Unattractive** |
| --- | --- | --- | --- |
| Me | Their | Beautiful | Ugly |
| Mine | Them | Good-Looking | Plain |
| My | They | Gorgeous | Hideous |
