# Supplementary material for: Implicit and explicit changes in body satisfaction evoked by body size illusions: Implications for eating disorder vulnerability in women
Source: PLoS One. 2018 Jun 21;13(6):e0199426. doi: 10.1371/journal.pone.0199426 (PMC6013093; doi:10.1371/journal.pone.0199426)
Supplement: S4 Table — Spearman’s Rho correlations for additional variables in experiment one. (DOCX) [file pone.0199426.s004.docx]

**S4 Table. Additional correlations.** Spearman’s Rho correlations for additional variables in experiment one. The p values are uncorrected.

|  | Eating Disorder Examination Questionnaire | | | | |  |  |  |  |  |  |  |
| --- | --- | --- | --- | --- | --- | --- | --- | --- | --- | --- | --- | --- |
|  | Global | Shape Concern | Weight Concern | Eating Concern | Restraint | Self-Esteem | Age | BMI | BMI diff Obese | BMI diff Slim | Obese ∆Body Satisfaction | Slim ∆Body Satisfaction |
| Global |  | *r_s_* = .940  p < .001 | *r_s_* = .924  p < .001 | *r_s_* = .558  p < .001 | *r_s_* = .697  p < .001 | *r_s_* = .132  p =.417 | *r_s_* = -.10  p = .539 | *r_s_* = .377  p = .017 | *r_s_* = -.425  p = .006 | *r_s_* = .468  p = .002 | *r_s_* = -.385*  p = .014 | *r_s_* = 315*  p = .047 |
| Shape Concern |  |  | *r_s_* = .832  p < .001 | *r_s_* = .519  p = .001 | *r_s_* = .556  p < .001 | *r_s_* = .76  p =.641 | *r_s_* = -.091  p = .575 | *r_s_* = .301  p = .059 | *r_s_* = -.358  p = .023 | *r_s_* = .348  p = .014 | *r_s_* = -.395  p = .012 | *r_s_* = .264  p = .105 |
| Weight Concern |  |  |  | *r_s_* = .487  p = .001 | *r_s_* = .547  p < .001 | *r_s_* = .697  p < .001 | *r_s_* = -.126  p =.437 | *r_s_* = .342  p = .031 | *r_s_* = -.371  p = .019 | *r_s_* = 371  p = .019 | *r_s_* = -.313  p = .049 | *r_s_* = -.32  p = .044 |
| Eating Concern |  |  |  |  | *r_s_* = .305  p =.055 | *r_s_* = .558  p < .001 | *r_s_* = -.118  p = .467 | *r_s_* = .153  p =.344 | *r_s_* = -.269  p = .093 | *r_s_* = .230  p = .153 | *r_s_* = -.151  p = .352 | *r_s_* = .421  p = .007 |
| Restraint |  |  |  |  |  | *r_s_* = .924  p < .001 | *r_s_* = .063  p = .701 | *r_s_* = .416  p = .008 | *r_s_* = -.340  p =.032 | *r_s_* = .448  p = .004 | *r_s_* = -.259  p = .106 | *r_s_* = -.037  p =821 |
| Self-Esteem |  |  |  |  |  |  | *r_s_* = .170  p = .294 | *r_s_* = .222  p = .168 | *r_s_* = .302  p = .058 | *r_s_* = .292  p =.068 | *r* = -.307  p = .054 | *r_s_* = .146  p = 368 |
| Age |  |  |  |  |  |  |  | *r_s_* = .046  p = .774 | *r_s_* = -.019  p = .909 | *r_s_* = .021  p = .896 | *r_s_* = -.051  p =.754 | *r_s_* = .118  p = .469 |
| BMI |  |  |  |  |  |  |  |  | *r_s_* = -.701  p < .001 | *r_s_* = .902  p < .001 | *r* = -.138  p = .396 | *r_s_* = .28  p = .08 |
| BMI diff Obese |  |  |  |  |  |  |  |  |  | *r_s_* = .928  p < .001 | *r_s_* = .175  p =.279 | *r_s_* = -.395  p = .012 |
| BMI diff Slim |  |  |  |  |  |  |  |  |  |  | *r_s_* = .163  p = .315 | *r_s_* = .363  p =.021 |
| Obese ∆Body Satisfaction |  |  |  |  |  |  |  |  |  |  |  | *r_s_* = -.356  p =.024 |
| Slim ∆Body Satisfaction |  |  |  |  |  |  |  |  |  |  |  |  |
