# Supplementary material for: Implicit and explicit changes in body satisfaction evoked by body size illusions: Implications for eating disorder vulnerability in women
Source: PLoS One. 2018 Jun 21;13(6):e0199426. doi: 10.1371/journal.pone.0199426 (PMC6013093; doi:10.1371/journal.pone.0199426)
Supplement: S5 Table — Spearman’s Rho and Pearson correlations for additional variables in experiment two: Sync vs Async = change in IAT d score for synchronous minus asynchronous trails (across body sizes). (DOCX) [file pone.0199426.s005.docx]

**S5 Table: Additional correlations.** Spearman’s Rho and Pearson correlations for additional variables in experiment two: Sync vs Async = change in IAT score for synchronous minus asynchronous trials (across body sizes). The p values are uncorrected.

|  | Eating Disorder Examination Questionnaire | | | | |  |  |  |  |  |  |  |  |
| --- | --- | --- | --- | --- | --- | --- | --- | --- | --- | --- | --- | --- | --- |
|  | Global | Shape Concern | Weight Concern | Eating Concern | Restraint | Self-Esteem | Age | BMI | BMI diff Obese | BMI diff Slim | Obese ∆Body Satisfaction | Slim ∆Body Satisfaction | Sync vs Async |
| Global |  | *r_s_* = .896  p < .001 | *r_s_* = .878  p < .001 | *r_s_* = .687  p < .001 | *r_s_* = .724  p < .001 | *r_s_* = -.421  p < .001 | *r_s_* = -.124  p = .328 | *r_s_* = -.01  p = .935 | *r_s_* = -.352  p = .004 | *r_s_* = .214  p = .089 | *r_s_* = -.342  p = .006 | *r_s_* = -.136  p = .283 | *r_s_* = -.341  p = .006 |
| Shape Concern |  |  | *r_s_* = .82  p < .001 | *r_s_* = .434  p < .001 | *r_s_* = .494  p < .001 | *r_s_* = -.486  p < .001 | *r_s_* = -.045  p = .723 | *r_s_* = -.022  p = .861 | *r_s_* = -.336  p = .007 | *r_s_* = .199  p = .115 | *r_s_* = -.319  p = .01 | *r_s_* = -.183  p = .149 | *r_s_* = -.339  p = .006 |
| Weight Concern |  |  |  | *r_s_* = .596  p < .001 | *r_s_* = .457  p < .001 | *r_s_* = -.454  p < .001 | *r_s_* = -.045  p =.722 | *r_s_* = .078  p = .538 | *r_s_* = -.372  p = .002 | *r_s_* = .256  p = .042 | *r_s_* = -.315  p = .011 | *r_s_* = -.110  p = .386 | *r_s_* = -.280  p = .025 |
| Eating Concern |  |  |  |  | *r_s_* = .589  p <.001 | *r_s_* = -.248  p = .061 | *r_s_* = -.213  p = .091 | *r_s_* = .003  p =.98 | *r_s_* = -.219  p = .082 | *r_s_* = .121  p = .342 | *r_s_* = -.176  p = .165 | *r_s_* = .008  p = .948 | *r_s_* = -.160  p = .207 |
| Restraint |  |  |  |  |  | *r_s_* = -.123  p =.357 | *r_s_* = .167  p = .188 | *r_s_* = .036  p = .776 | *r_s_* = -.330  p =.008 | *r_s_* = .217  p =.085 | *r_s_* = -.178  p = .159 | *r_s_* = -.056  p =.662 | *r_s_* = -.175  p = .167 |
| Self-Esteem |  |  |  |  |  |  | *r_s_* = .170  p = .203 | *r_s_* = .007  p = .96 | *r_s_* = .226  p = .088 | *r_s_* = -.134  p = .316 | *r_S_* = .128  p = .339 | *r_s_* = -.103  p = .443 | *r_s_* = -.019  p = .890 |
| Age |  |  |  |  |  |  |  | *r_s_* = .015  p = .904 | *r_s_* = -.009  p = .924 | *r_s_* = .008  p = .947 | *r_s_* = .188  p =.136 | *r_s_* = .023  p = .858 | *r_s_* = .176  p = .163 |
| BMI |  |  |  |  |  |  |  |  | *r_s_* = -.677  p < .001 | *r_s_* = .873  p < .001 | *r* = .254  p = .051 | *r_s_* = .014  p = .913 | *r* = .190  p = .133 |
| BMI diff Obese |  |  |  |  |  |  |  |  |  | *r_s_* = -.941  p < .001 | *r_s_* = .014  p =.931 | *r_s_* = -.003  p = .98 | *r_s_* = .097  p = .447 |
| BMI diff Slim |  |  |  |  |  |  |  |  |  |  | *r_s_* = .104  p = .415 | *r_s_* = .005  p =.967 | *r_s_* = .172  p = .174 |
| Obese ∆Body Satisfaction |  |  |  |  |  |  |  |  |  |  |  | *r_s_* = -.028  p =.827 | *r* = .757  p < .001 |
| Slim ∆Body Satisfaction |  |  |  |  |  |  |  |  |  |  |  |  | *r_s_* = .620  p < .001 |
