# Supplementary material for: Implicit and explicit changes in body satisfaction evoked by body size illusions: Implications for eating disorder vulnerability in women
Source: PLoS One. 2018 Jun 21;13(6):e0199426. doi: 10.1371/journal.pone.0199426 (PMC6013093; doi:10.1371/journal.pone.0199426)
Supplement: S1 Text — Additional results for questionnaire items in experiments one and two. (DOCX) [file pone.0199426.s006.docx]

**S1 text: Supplementary Results**

**Ownership for females and males independently**

**Experiment one**

To directly assess differences in illusion strength between the different body sizes, Wilcoxon signed rank tests were conducted between obese and slim bodies for the synchronous trials only, which indicated no significant difference (z  =  -1.47, p = .238, r = .16). Mann-Whitney U tests demonstrated that illusion scores were equivalent for the male and female participants for both the slim (z  =  -.384, p = .718, r = .04) and obese (z  =  −.466, p = .659, r = .05) bodies.

In independent examinations of the male and female illusion scores, Wilcoxon signed rank tests indicated significantly higher scores for the synchronous trials for both body sizes with both male and female participants (female slim z = -3.67, p < .001; female obese z = -3.67, p = .018; male slim z = -3.01, p = .009; male obese z = -3.54, p < .001).

No significant differences were identified between the synchronous and asynchronous trials for the control questions (female slim z = -.673, p = 501; female obese z = -1.14, p = .381; male slim z = -.489, p = .625; male obese z = -.925, p = .533).

There were also no significant differences in the illusion strength between the different body sizes for the synchronous and asynchronous trials for either sex (female synchronous z = -1.36, p = .348; female asynchronous z = -.808, p = 501; male synchronous z = -.745 p = .456; male asynchronous z = -2.22, p = .054).

**Experiment two**

Mann-Whitney U tests demonstrate that the illusion scores were equivalent for the male and female participants for both the slim (synchronous z  =  -.320, p = .749, r = .04, asynchronous z  =  -1.28, p = .749, r = .16) and obese (synchronous z  =  -1.8, p = .164, r = .23, asynchronous z  =  -1.74, p = .164, r = .22) bodies.

In independent examinations of the male and female illusion scores, Wilcoxon signed rank tests indicated significantly higher scores for the synchronous trials for both body sizes with both male and female participants (female slim z = -4.56, p < .001; female obese z = -4.15, p < .001; male slim z = -4.28, p < .001; male obese z =-3.70, p < .001).

No significant differences were identified between the synchronous and asynchronous trials for the control questions (female slim z = -.549, p = .875; female obese z = -.26, p = .914; male slim z = -1.46 p = .175; male obese z = -.707, p = .48).

There were no significant differences in the illusion strength between the different body sizes for the synchronous or asynchronous trials for the female participants (synchronous z = -.11, p = .914; asynchronous z = -.64, p = .875). However, for the males, the slim conditions produced stronger agreement for both the synchronous (slim median = 2; obese median = 1; z = -2.39, p = .026) and asynchronous (slim median = -.5; obese median = -1.75; z = -3.37, p = .002) trials. These p values were all adjusted for multiple comparisons using false discovery rate (FDR) correction.

**Summary**

Overall, these results show that the illusion experience was successful for both males and females with both body sizes. For females, feelings of ownership were statistically equivalent for slim and obese bodies. For males, ownership was stronger for the slim bodies than for the obese bodies. However, for all synchronous conditions, the median responses were +1 and demonstrated the illusion was successful on a group level for males and females with both slim and obese bodies.

**Attractiveness ratings**

**Experiment one**

We analysed the questionnaire ratings for the statement “The body in the image was attractive”. Across the entire sample, there was no significant difference between the synchronous and asynchronous trials for the slim condition (z = -.367, p = .937) or the obese condition (z = -.262, p = .937). When the synchronous trials only were examined, agreement was stronger with the slim (median = 1) than obese (median = -2) bodies (z = -5.09, p < .001).

For females, there was no significant difference between the synchronous and asynchronous trials for the slim condition (z = -.106, p = .943) or the obese condition (z = -.072, p = .943). When the synchronous trials only were examined, agreement in the slim (median = 1) condition was greater than that in the obese (median = -1.5) condition (z = -3.22, p = .004).

For males, there was no significant difference between the synchronous and asynchronous trials for the slim condition (z = -.371, p = .937) or the obese condition (z = -.333, p = .937). When the synchronous trials only were examined, agreement in the slim (median = 2) condition was greater than that in the obese (median = -2.5) condition (z = -3.86, p < .001).

When comparing males and females directly, there were no significant differences for the conditions (obese synchronous, z = -2.28, p = .072; obese asynchronous, z = -1.81, p = .182; slim synchronous, z = -1.556, p = .260; slim asynchronous, z = -1.05, p = .546). All p values are FDR corrected.

Moreover, attractiveness ratings were not correlated with body satisfaction scores in the synchronous conditions (slim entire sample, r = .145, p = .690, slim male r = 326, p = .690; slim female, r = .017, p = .943; obese entire sample, r = . 318, p = .690; obese male, r = .123, p = .726; obese female, r = .463, p = .690) or relative to baseline (slim entire sample, r = .200, p = .690; slim male r = .076, p = .814; slim female, r = .242, p = .690; obese entire sample, r = .333, p = .690; obese male, r = .146, p = .718; obese female, r = .461, p = .690) for either body size. All p values are FDR corrected.

**Experiment two**

Across the entire sample, there was no significant difference between the synchronous and asynchronous trials for the slim condition (z = -.796, p = .554) or the obese condition (z = -.024, p = .981). When the synchronous trials only were examined, agreement was higher for the slim (median = 1) than obese (median = -2) bodies (z = -5.76, p < .001).

For the females, there was no significant difference between the synchronous and asynchronous trials for the slim condition (z = -.117, p = .981) or the obese condition (z = -1.04, p = .489). When the synchronous trials only were examined, agreement for the slim (median = 1) body was greater than for the obese (median = -2) body (z = -3.32, p = .004).

For the males, there was no significant difference between the synchronous and asynchronous trials for the slim condition (z = -1.34, p = .388) or the obese condition (z = -.893, p = .537). When the synchronous trials only were examined, agreement for the slim (median = .5) body was greater than for the obese (median = -2) body (z = -4.73, p < .001). All p values were adjusted for multiple comparisons using FDR correction.

When comparing males and females directly, there was a significant difference in the agreement for the obese body synchronous condition (z = -2.59, p = .029). The median scores for the males and females were both -2; however, the interquartile range indicated more negative results for the males (1st quartile = -3, 3^rd^ quartile = -2) than for the females (1st quartile = -2, third quartile = 0). No significant differences were identified for the other conditions (obese asynchronous, z = -2.02, p = .114; slim synchronous, z = -.185, p = .981; slim asynchronous, z = -1.04, p = .489). All p values are FDR corrected.

Furthermore, attractiveness ratings were not correlated with body satisfaction scores in the synchronous conditions (slim entire sample, r = .007, p = .985, slim male r = -.235, p = .648; slim female, r = .116, p = .840; obese entire sample, r = -.09, p = .840; obese male, r = .119, p = .840; obese female, r = .225, p = .648) or relative to baseline (slim entire sample, r = .034, p = .946; slim male r = -.003, p = .985; slim female, r = .094, p = .840; obese entire sample, r = -.061, p = .840; obese male, r = .237, p = .648; obese female, r = -.294, p = .6648) for either body size. All p values are FDR corrected.

**Summary**

Overall, these results demonstrate that participants, on average, found the slim body to be more attractive than the obese body, and this finding was not modulated by the illusion for females or males. Attractiveness ratings were broadly similar for males and females, and attractiveness was not related to body satisfaction scores.
